# In Vitro Characterization and Stability Profiles of Antibody–Fluorophore Conjugates Derived from Interchain Cysteine Cross-Linking or Lysine Bioconjugation

**DOI:** 10.3390/ph12040176

**Published:** 2019-12-02

**Authors:** Camille Martin, Guillaume Brachet, Cyril Colas, Emilie Allard-Vannier, Claire Kizlik-Masson, Clara Esnault, Renaud Respaud, Caroline Denevault-Sabourin, Igor Chourpa, Valérie Gouilleux-Gruart, Marie-Claude Viaud-Massuard, Nicolas Joubert

**Affiliations:** 1 GICC EA7501, Equipe IMT, Université de Tours, UFR des Sciences Pharmaceutiques, 31 Avenue Monge, 37200 Tours, France; camille.martin-4@etu.univ-tours.fr (C.M.); clara.esnault@etu.univ-tours.fr (C.E.); caroline.denevault@univ-tours.fr (C.D.-S.); mcviaud@univ-tours.fr (M.-C.V.-M.); 2 Laboratoire d’immunologie, CHRU Tours, 10 Bd Tonnelé, 37000 Tours, France; guillaume.brachet@univ-tours.fr (G.B.); valerie.gouilleux@univ-tours.fr (V.G.-G.); 3 GICC EA7501, Equipe FRAME, Université de Tours, 10 Bd Tonnelé, 37000 Tours, France; claire.kizlik-masson@etu.univ-tours.fr; 4 ICOA UMR 7311 CNRS, Université d’Orléans, Pôle de Chimie, Rue de Chartres, BP 6759, 45067 Orléans CEDEX 2, France; cyril.colas@univ-orleans.fr; 5 CBM, UPR4301 CNRS, Rue Charles Sadron, 45071 Orléans, France; 6 NMNS EA 6295, 31 Avenue Monge, 37200 Tours, France; emilie.allard@univ-tours.fr (E.A.-V.); igor.chourpa@univ-tours.fr (I.C.); 7 INSERM U1100, Université de Tours, 10 Bd Tonnelé, 37000 Tours, France; renaud.respaud@univ-tours.fr

**Keywords:** antibody–fluorophore conjugate, fluorescence, labelling, bioconjugation

## Abstract

Fluorescent labelling of monoclonal antibodies (mAbs) is classically performed by chemical bioconjugation methods. The most frequent labelling technique to generate antibody–fluorophore conjugates (AFCs) involves the bioconjugation onto the mAb lysines of a dye bearing an *N*-hydroxysuccinimide ester or an isothiocyanate group. However, discrepancies between labelling experiments or kits can be observed, related to reproducibility issues, alteration of antigen binding, or mAb properties. The lack of information on labelling kits and the incomplete characterization of the obtained labelled mAbs largely contribute to these issues. In this work, we generated eight AFCs through either lysine or interchain cysteine cross-linking bioconjugation of green-emitting fluorophores (fluorescein or BODIPY) onto either trastuzumab or rituximab. This strategy allowed us to study the influence of fluorophore solubility, bioconjugation technology, and antibody nature on two known labelling procedures. The structures of these AFCs were thoroughly analyzed by mass spectroscopy, and their antigen binding properties were studied. We then compared these AFCs in vitro by studying their respective spectral properties and stabilities. The shelf stability profiles and sensibility to pH variation of these AFCs prove to be dye-, antibody- and labelling-technology-dependent. Fluorescence emission in AFCs was higher when lysine labelling was used, but cross-linked AFCs were revealed to be more stable. This must be taken into account for the design of any biological study involving antibody labelling.

## 1. Introduction

Fluorescent labelling of biomolecules is highly valuable for the study of biological processes in a sensitive, qualitative, or quantitative way. Through their specificity for a cognate antigen, chemically modified monoclonal antibodies (mAbs) [1] make powerful molecular tools for various applications [1,2,3]. Indeed, labelled mAbs can facilitate detection of targeted biological markers. They are also classically used as secondary antibodies for flow cytometry, enzyme-linked immunosorbent assay (ELISA) or immunofluorescence assay. Moreover, labelled mAbs with tunable fluorescent sensor dyes [4] or near-infrared dyes (NIR dyes) [3,5] have been used respectively to study molecular mechanisms, for in vivo imaging, and even for guided cancer surgery in human [6].

**Scheme 1:**
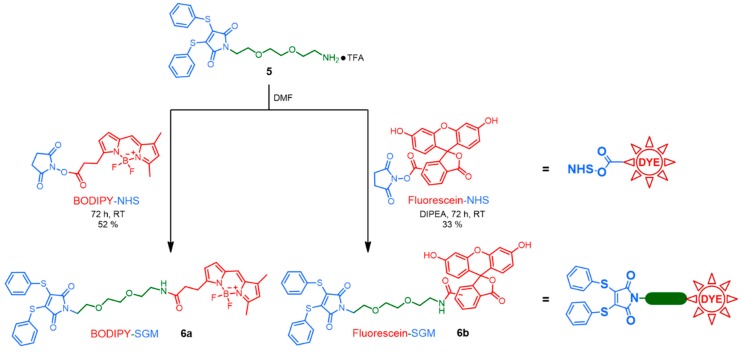
Synthesis of compounds designed for bioconjugation from precursor **5**: BODIPY-SGM **6a** and Fluorescein-SGM **6b**. Abbreviations: TFA = trifluoroacetic acid, NHS = *N*-hydroxysuccinimide, SGM = second generation maleimide, DIPEA = *N*,*N*-diisopropyléthylamine.

**Scheme 2:**
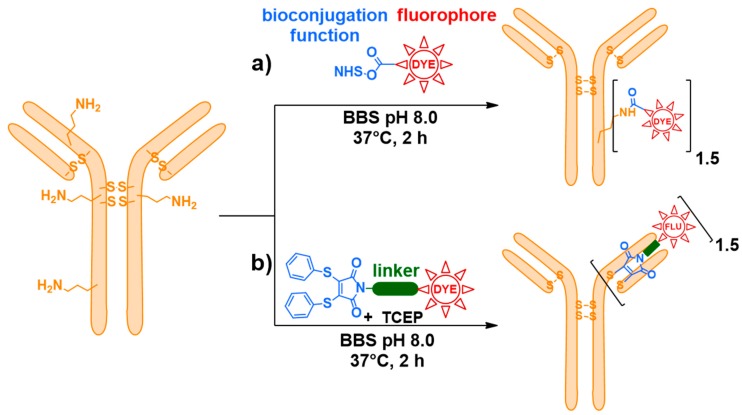
Bioconjugation reactions. (**a**) bioconjugation onto a native mAb (lysines) using *N*-hydroxysuccinimide (NHS) ester fluorophore resulting in a randomly functionalized AFC with an average of 1.5 fluorophores per mAb. (**b**) Bioconjugation onto a native mAb (disulfide bridges) using an SGM fluorogenic linker **6** and giving a cross-linked AFC with an average of 1.5 fluorophores per mAb. Abbreviations: BBS = borate buffer saline; TCEP = tris(2-carboxyethyl)phosphine; FLU = fluorescein.

Antibody–fluorophore conjugates (AFCs) are classically generated by chemical conjugation methods onto amino acids [2,7] using fluorescent dyes functionalized with reactive moieties allowing covalent attachment to amino acid lateral chains of the antibody. Amine, sulfhydryl, carbohydrate, or disulfide bonds are among the most favored reactive moieties for bioconjugation. Commercially available fluorescent labelling kits contain dyes bearing an *N*-hydroxysuccinimide (NHS) ester (Scheme 1) or an isothiocyanate function, permitting bioconjugation onto the ε-amino group of solvent-accessible lysines at the mAb surface (Scheme 2a). Up to 30 lysines can be randomly labelled using these methods [8], leading to heterogeneous mixtures of AFCs with varying numbers of fluorophores attached at different positions on the mAb, determining the FAR (fluorophore-to-antibody ratio). This method remains the most widely used for labelling. However, the lack of information provided within labelling kits contributes to these issues. Generally, the manufacturers invoke intellectual property issues to retain technological information regarding both the dye concentration and the buffer composition. As a consequence, it is impossible to estimate the number of conjugated dyes per labelled mAb and their localization on the protein. Moreover, conjugated lysines in the complementary determining region (CDR) or next to essential residues for Fc receptor (FcR) binding can respectively alter the affinity to antigen or effector properties of the labelled mAb [9,10,11]. The field of antibody–drug conjugate (ADC) research has widely described that conjugation site has a great impact on ADC physico-chemical properties including bond stability between the conjugated molecule and the mAb [12]. In this context, two recent publications compared various labelling methods [13,14]. While the authors confirmed previously described advantages and drawbacks associated with lysine conjugation, cysteine labelling also came with limitations. Even though first-generation maleimide (or FGM) [13] allows for covalent linkage to the cysteines of a reduced interchain disulfide bridge (mild reduction using either dithiothreitol (DTT) or tris(2-carboxyethyl) phosphine (TCEP)), it is relatively unstable, [15] undergoing a retro-Michael addition in aqueous or serum media, resulting in payload loss over time. This phenomenon could impair the stability of labelled mAbs, compromising biological studies employing them as biological tools. To challenge lysine conjugation, an alternative labelling method should be easy to use, simple, fast, and allow for a reproducible FAR.

In this context, our team [16,17,18] and several others [8,19] developed second-generation maleimides or SGM (dibromomaleimide or dithiophenylmaleimide) [16,20] to produce ADCs or AFCs. After mild reduction of the mAb, these SGMs are able to undergo bioconjugation onto mAb through interchain cysteine cross-linking (Scheme 2b) to afford stable AFCs with a controlled FAR.

In this paper, we report the construction and characterization of eight labelled mAbs, resulting from the conjugation of trastuzumab and rituximab with two commercial green fluorophores using two well-known conjugation methods. Cross-linked mAbs were compared to their labelled counterparts resulting from NHS conjugation in order to evaluate the discrepancies between their respective fluorescence emissions, stability upon storage, or pH shift. To avoid aggregation, [21] labelled mAbs were produced with an average FAR of 1.5.

## 2. Materials and Methods

Unless otherwise noted, materials were obtained from commercial suppliers at the highest purity grade available and used without further purification. Dry solvents were purchased from Acros Organics (Fisher Scientific SAS, Illkirch, France), other solvents were purchased from Carlo Erba Reagents (Val-de-Reuil, France). BODIPY-NHS ester was purchased from Lumiprobe Gmbh. Fluorescein-NHS ester was purchased from ThermoFisher (Illkirch, France). Other reagents were purchased from Sigma-Aldrich (Merck, Darmstadt, Germany).

For anhydrous and inert reactions, the glassware was heated with a heat gun while several vacuum-dry argon cycles were performed. The thin layer chromatography (TLC) studies were performed using commercial pre-coated aluminum sheets of silica gel (60 Å, F_254_; Merck) and revealed under UV 254 and 365 nm lighting. The purifications by chromatography on silica gel columns were carried out on a TELEDYNE ISCO (Lincoln, NE, USA) purification unit, Combi Flash RF 75 PSI, with Redisep flash silica gel columns (60 Å, 230–400 mesh, grade 9385). Nuclear Magnetic Resonance (NMR) spectra were measured on a Bruker (Bremen, Germany) Ultrashield 300 spectrometer, 300 MHz (^1^H), 282 MHz (^19^F) and 75 MHz (^13^C). Chemical shifts are reported in parts per million (ppm, δ), downfield from tetramethylsilane (TMS, δ = 0.00 ppm), and are referenced to the residual solvent. Coupling constants are reported in Hertz (Hz). The following abbreviations are used singularly or in combination to indicate the multiplicity of signals: s—singlet, d—doublet, t—triplet, q—quartet, m—multiplet. NMR spectra were acquired at 300 K unless otherwise indicated. High-resolution accurate mass measurements (HRAM) were performed in positive mode with an electrospray ionization (ESI) source on a Q-TOF DataAnalysis 4 maXis mass spectrometer (Bruker, Bremen, Germany) with an accuracy tolerance of 2 ppm by the “Fédération de Recherche” ICOA/CBM (FR2708) platform.

### 2.1. Synthesis

Synthesis of compounds 1, 2, 3, 4, and 5 are described in the supporting information (Scheme S1).

#### 2.1.1. 3,4-Dithiophenoylmaleimide-*N*-8-amino-3,6-dioxaoctane-BODIPY **6a**

**5** (12 mg, 0.021 mmol) was dissolved in dry dimethylformamide (DMF) (400 µL) and put in a 1.5 mL vial with BODIPY-NHS (9.9 mg, 0.025 mmol) under inert atmosphere. It was stirred in the dark at rt for 3 days. The crude mixture was diluted with ethyl acetate (10 mL) and washed with brine at 0 °C (5 × 10 mL). Combined organic phases were dried over MgSO_4_, filtered, and concentrated under reduced pressure. It was purified by flash chromatography (SiO_2_, cyclohexane/ethyl acetate 7:3) to give **6a** (7.9 mg, 52%) as an orange oil. ^1^H NMR (300 MHz, CDCl_3_) δ (ppm) 7.32–7.13 (m, 11H, NH(CO), Ph), 7.06 (s, 1H, CH Ar), 6.85 (d, *J* = 3.8 Hz, 1H, CH Ar), 6.29 (d, *J* = 3.8 Hz, 1H, CH Ar), 6.10 (s, 1H, CH Ar), 3.68 (t, *J* = 5.3 Hz, 2H, CH_2_), 3.59 (t, *J* = 5.3 Hz, 2H, CH_2_-O), 3.55–3.52 (m, 2H, CH_2_–O), 3.49–3.39 (m, 6H, CH_2_–O, CH_2_–NH(CO)), 3.28 (t, *J* = 7.5 Hz, 2H, CH_2_–(CO)), 2.63 (t, *J* = 7.5 Hz, 2H, CH_2_), 2.54 (s, 3H, CH_3_), 2.24 (s, 3H, CH_3_). ^13^C NMR (75 MHz, CDCl_3_) δ (ppm) 172.1, 167.0 (×2), 160.1, 158.1, 143.7, 135.8, 133.5, 132.0 (×4), 129.1 (×4), 128.5 (×4), 123.8, 120.4, 117.7, 77.6, 77.4, 77.2, 76.7, 70.5, 70.0, 69.8, 67.8, 39.4, 38.1, 35.8, 24.9, 15.1, 11.5. ^19^F (282 MHz, CDCl_3_) δ (ppm) –145 (q, *J* = 33.9 Hz). HRAM (ESI): *m/z* calc. = 719.2339, found 719.2362.

#### 2.1.2. 3,4-Dithiophenoylmaleimide-*N*-8-amino-3,6-dioxaoctane-FLU **6b**

**5** (27 mg, 0.048 mmol) was dissolved in dry DMF (1 mL) and put on FLU-NHS (23 mg, 0.048 mmol) under inert atmosphere. Diisopropylethylamine (8.4 µL, 0.048 mmol) was added and it was stirred in the dark at rt for 3 days. The crude was diluted with CH_2_Cl_2_ (10 mL) and washed with brine at 0 °C (5 × 10 mL). Combined organic phases were dried over MgSO_4_, filtered, and concentrated under reduced pressure. It was purified by flash chromatography (SiO_2_, CH_2_Cl_2_/MeOH 9:1) to give **6b** (13 mg, 33%) as an orange oil. ^1^H NMR (300 MHz, MeOD)* δ (ppm) 8.39 (s, 0.5H, Ar), 8.14 (dd, *J* = 8.1, 1.6 Hz, 0.5H, Ar), 8.04–8.00 (m, 0.5H, Ar), 7.68 (s, 0.5H, Ar), 7.16–7.07 (m, 10H, Ph), 6.68–6.57 (m, 2H, Ar), 6.56–6.49 (m, 2H, Ar), 6.49–6.41 (m, 2H, Ar), 3.70–3.49 (m, 6H, CH_2_, CH_2_–O), 3.49–3.36 (m, 4H, CH_2_–O), 3.36–3.30 (m, 2H, CH_2_–NH(CO)). ^13^C NMR (75 MHz, MeOD) δ (ppm) 170.6, 168.4, 168.1, 161.5, 154.1, 142.1, 137.7, 137.0, 135.5, 132.5, 132.4, 130.7, 130.6, 130.6, 130.5, 130.3, 130.12, 130.1, 129.3, 129.2, 126.1, 125.8, 125.0, 124.3, 113.8, 110.9, 103.6, 71.3, 71.1 (×2), 70.8, 70.4, 70.3, 68.7, 68.7, 41.2, 41.0, 39.2, 39.2. HRAM (ESI): *m/z* calc. = 803.1728, found 803.1718.

* Both isomers are described.

### 2.2. Bioconjugation

TTZ and RTX were prepared in borate buffer (400 µL, 4.5 mg/mL) at pH 8.0. Then 18.0 µL of a dimethylsulfoxyde (DMSO) solution of linkers **6a** or **6b** (15 eq) was added. DMSO volume was corrected to 10% v/v, and 7.2 µL of a freshly prepared solution of TCEP in borate buffer pH 8.0 (6 eq) was added. It was gently shaken under inert atmosphere for 2 h at 37 °C, yielding AFCs **7a**, **7b**, **8a,** and **8b**. Their lysine counterparts were prepared with DMSO solutions of NHS ester dyes (2 to 5 eq) gently shaken with mAbs for 2 h at 37 °C, yielding AFCs **9a**, **9b**, **10a**, and **10b**.

Crude AFCs were purified by gel filtration using Sephadex G-25 (Fisher Scientific SAS, Illkirch, France) against phosphne buffer saline (PBS) 1X pH 7.2 and filtered on 0.22 µm membranes. The protein concentration of purified AFCs was assessed by UV absorption at 280 nm (Nanodrop, Fisher Scientific SAS, Illkirch, France).

### 2.3. Mass Spectrometry

Mass spectrometric analyses of AFCs were performed on a Bruker maXis mass spectrometer coupled to a Dionex Ultimate 3000 RSLC system (Dionex, Germering, Germany). Prior to mass spectrometry (MS) analysis, samples (ca. 5 µg) were desalted on a MassPREP (Waters, Saint-Quentin-en-Yvelines, France) desalting cartridge (2.1 × 10 mm, Waters) heated at 80 °C using 0.1% formic acid as solvent A and 0.1% formic acid in acetonitrile as solvent B at 500 µL/min. After 1 min, a linear gradient from 5 to 90% B in 1.5 min was applied; the first 1.5 min were diverted to waste. MS data were acquired in positive mode with an ESI source over the m/z range from 900 up to 5000 at 1 Hz and processed using DataAnalysis 4.4 software (Bruker, Bremen, Germany) and the MaxEnt algorithm for spectral deconvolution.

### 2.4. HER2 Binding by ELISA

The functionality of AFCs was checked by indirect ELISA using the HER2 protein (Sino Biologicals, Beijing, China) as a target. The samples were detected by protein L-peroxydase (Thermo Scientific Pierce, Illkirch, France) in the presence of a chromatic substrate, 3,3’,5,5’-tetramethylbenzidine (TMB; Sigma, St. Louis, MO, USA). Briefly, HER2 was coated in a 96-well plate at 1 µg/mL and incubated overnight at 4 °C. The wells were then saturated with 3% bovine serum albumin in phosphate buffer saline (BSA–PBS) for 1 h at 37 °C and washed with PBS prior to incubation with AFC from 0.01 nM to 31.00 nM during 1 h at 37 °C. Wells were then washed with PBS–tween 20 (0.05%) and incubated with 100 µL of protein-L-peroxydase (1.25 µg/mL) for 1 h at 37 °C added to 100 µL of TMB substrate (Sigma-Aldrich, St. Louis, MO, USA). Enzymatic reactions were stopped with the addition of 50 µL of 1M H_2_SO_4_, and the absorbance was measured at 450 nm using a microplate reader (Biotek, Winooski, VT, USA).

### 2.5. CD20 Binding by Flow Cytometry

Daudi cells were obtained from American Type Culture Collection (ATCC, CCL-213™). Daudi cells were harvested and successively washed in Roswell Park Memorial Institute (RPMI) and Hank’s Balanced Salt Solution (HBSS) media. Cell count was adjusted to 2 × 10^6^ cells/mL in HBSS buffer. The Daudi cells (5 × 10^4^ cells) were incubated for an hour at 4 °C in the dark with the various ADCs diluted at 0, 1, 3, 10, 30, or 100 µg/mL in HBSS buffered either at pH 6 (with 2-(*N*-morpholino) ethanesulfonic acid (MES)) or 7. They were then washed with HBSS and immediately processed on a 2-laser-8-colour Gallios flow cytometer (Beckman Coulter, Brea, CA, USA). Ten thousand events were acquired per condition by flow cytometry in the Gallios List Mode Data Acquisition and Analysis Software 1.2. Raw data from all samples were analyzed using Kaluza Analysis Software v.1.1.11052.10190 (Beckman Coulter). A gate was set on living cells using the forward scatter–side scatter plot. The ratio of mean fluorescence at each concentration versus cells alone was used to establish the fluorescence curves. All flow cytometry experiments were performed three times independently.

### 2.6. Fluorescence Emission

Fluorescence emission spectra of the AFC solutions in PBS 1X pH 7.2 buffer were recorded from 295 to 700 nm with a Hitachi F-4500 fluorescence spectrophotometer. Excitation was set to 275 nm to minimize Raman scattering, which can interfere with fluorescence emission at weak concentrations while being energetic enough to see the fluorescence emission of both antibody and dye. Concentration in protein was adjusted to 50 µg/mL, and spectra were normalized at the intensity of the tryptophan (Trp) band at 350 nm before comparison.

## 3. Results and Discussion

We aimed at designing a reproducible labelling process affording all AFCs with the same FAR to be comparable. For this purpose, we purchased two commercially available green-emitting fluorophores (Scheme 1), fluorescein-NHS and BODIPY-NHS, as solid powder and not as a labelling kit, allowing us to prepare DMSO solutions with known concentrations to control the bioconjugation step. These compounds have fluorescence emission maxima at similar wavelengths (520 nm and 509 nm, respectively) with high quantum yields (0.90 and 0.97, respectively). Although more photostable dyes could have been used in this study, we chose fluorescein and BODIPY common dyes of different size and solubility to better observe discrepancies in the assessment of the stability profiles of our AFCs, which make them good candidates for our comparative labelling study. From Fluorescein and BODIPY, since they were functionalized with NHS-ester to allow conjugation on lysines (Scheme 2a), we synthesized the corresponding dithioether SGM-bearing dyes (Scheme 1) to perform an interchain cysteine cross-linking reaction onto mAbs (Scheme 2b). We chose the anti-HER2 trastuzumab (TTZ) and the anti-CD20 rituximab (RTX) as antibody carriers (IgG1κ) to analyze potential antibody-dependent discrepancies during the bioconjugation process with two different well-known bioconjugation method. FAR value should be limited to avoid AFC aggregation and the loss of mAb biological properties [21]. Therefore, we produced all labelled mAbs (Table 1) with an average FAR of 1.5 verified through mass spectroscopy analysis. We studied binding to antigen for all AFCs before comparing their stability profiles under various conditions.

### 3.1. Synthesis of Linkers

2,3-Dibromomaleimide was efficiently activated with methylchloroformiate to afford precursor **1**. In the presence of a short polyethylene glycol chain monoprotected with a *tert*-butyloxycarbonyl group **2, 1** afforded intermediate **3** according to a previously described synthesis [22]. Deprotection of **3** using TFA to obtain **4**, followed by conversion of bromines into phenylthioethers, gave the trifluoroacetic salt **5** with a 43% yield over 4 steps (Scheme S1).

BODIPY-NHS and Fluorescein-NHS, in the presence of compound **5,** afforded the desired BODIPY-SGM **6a** and Fluorescein-SGM **6b,** respectively (Scheme 1). Fluorescein-NHS was less reactive and necessitated the addition of *N*,*N*-diisopropyléthylamine (DIPEA) to react on **5**.

### 3.2. Bioconjugation onto mAbs

It is known that heavily loaded mAbs aggregate or lose function [23,24,25]. To avoid these problems, we targeted a FAR of 1.5 to limit AFC aggregation.

First, we conjugated TTZ and RTX onto disulfide bridges to linkers BODIPY-SGM **6a** or Fluorescein-SGM **6b** to yield, respectively, AFCs TTZ-SGM-BDP **7a**, TTZ-SGM-FLU **7b**, RTX-SGM-BDP **8a,** and RTX-SGM-FLU **8b** (Table 1, Scheme 2b). We managed to obtain an average FAR of 1.5 for each cross-linked AFC with 6 eq of TCEP and 15 eq of linkers per antibody.

Then, TTZ and RTX were conjugated on lysines to BODIPY-NHS ester or to fluorescein-NHS ester to afford, respectively, AFCs TTZ-BDP **9a**, TTZ-FLU **9b**, RTX-BDP **10a**, and RTX-FLU **10b** (Table 1, Scheme 2a). For both TTZ and RTX, while BDP-NHS required around 3 eq per mAb to achieve the desired FAR of 1.5, FLU-NHS necessitated 10 eq per mAb to reach the same result (Table S1).

### 3.3. Mass Spectrometry

**Figure 1:**
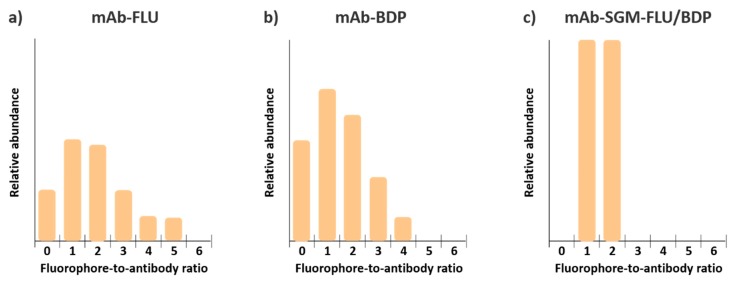
Relative abundance of labelled antibodies in each AFC according to their fluorophore-to-antibody ratio (FAR): (**a**) mAb-FLU **9b** and **10b**; (**b**) mAb-BDP **9a** and **10a**; (**c**) mAb-SGM-FLU/BDP **7a**, **7b**, **8a,** and **8b**. The average FAR is 1.5 for all AFCs. mAb = TTZ or RTX. Abbreviations: BDP = BODIPY; FLU = fluorescein; TTZ = trastuzumab; RTX = rituximab.

Mass spectrometry analysis was used to determine the FAR distribution of all AFCs (Tables S2 and S3). We used an electrospray ionization (ESI) source, producing multicharged ions. Compared to matrix-assisted laser desorption/ionization (MALDI) technology, which produces monocharged species, ESI sources improve resolution (Figures S1–S3). Careful study of mass spectra allowed us to determine the FAR distribution and, subsequently, the average FAR of each AFC (Figure 1). As shown in Figure 1, all tested AFCs had an average FAR of 1.5 calculated from the FAR distribution (Figure 1). As expected, for the same average FAR, the distribution of SGM-based AFCs **7a**, **7b**, **8a,** and **8b** was more homogeneous (2 species, FAR 1 and 2) compared to their lysine-conjugated counterparts **9a**, **9b**, **10a,** and **10b** (5 or 6 species, ranging from FAR 0 to FAR 5) [16,26,27].

### 3.4. Antigen Binding

As the chemical modification of mAbs should have minimal interference on antigen binding, we determined HER2 affinities of AFCs TTZ-SGM-FLU **7b** and TTZ-FLU **9b** by ELISA (Figure S4). We observed no difference between the two AFCs, with similar affinity to HER2 for AFC generated through either interchain cystein cross-linking or lysine bioconjugation.

**Figure 2:**
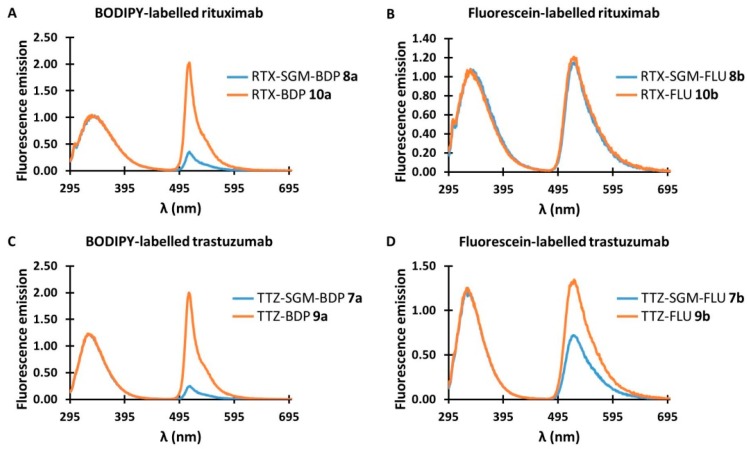
Comparison of fluorescence emissions of cross-linked AFCs (blue curves) and lysine-functionalized AFCs (orange curves) after normalization: (**A**) BDP-labelled RTX; (**B**) FLU-labelled RTX; (**C**) BDP-labelled TTZ; (**D**) FLU-labelled TTZ. Lower and higher wavelength bands correspond to the intrinsic emission of antibody aromatic amino acids (mainly tryptophan) and conjugated fluorophore emission, respectively. The excitation wavelength was 275 nm. For easier comparison, the sample concentration was adjusted to 50 µg/mL of protein.

In parallel, we assessed the binding of AFCs RTX-SGM-FLU **8b** and RTX-FLU **10b** to CD20 using flow cytometry (Figures S5–S7). Considering their fluorescence emission data (Figure 2B), it is possible to compare directly the binding to CD20 antigen expressed on Daudi cells via fluorescence ratios (Figures S5 and S7). Flow cytometry experiments showed a significantly higher fluorescence ratio for AFC RTX-SGM-FLU **8b** as compared to AFC RTX-FLU **10b** at pH 7 (Figure S5, Table S4). Hence, AFC RTX-SGM-FLU **8b** had a higher binding to CD20 than AFC RTX-FLU **10b** at pH 7.

### 3.5. Comparative Fluorescence Emission between AFCs

One of the common drawbacks of fluorescent protein labelling is the modification of the dye quantum yield occurring upon labelling [23,28,29]. Therefore, we measured and compared the fluorescence emission of AFCs of several mAb–dye pairs to evaluate the effect of the labelling method on the dye fluorescence properties (Figure 2). We first measured UV-visible absorption of AFCs (Figure S8). We set the excitation wavelength at 275 nm in order to be sufficiently energetic to allow the observation of fluorescence emission from both the dye and mAb. We observed the fluorescence emissions of both moieties in the AFCs, respectively, at higher and lower wavelength bands, the latter being due to tryptophans (Trps) inside the mAb amino acid sequence. The Trp emission did not change significantly between comparable samples (neither in intensity nor in the spectral shape); therefore, it was used as an internal intensity standard.

The BODIPY fluorescence emission was strongly quenched in TTZ-SGM-BDP **7a** and RTX-SGM-BDP **8a** compared, respectively, to AFCs TTZ-BDP **9a** and RTX-BDP **10a**. For the fluorescein dye, there was no difference in the fluorescence emission between RTX-SGM-FLU **8b** and RTX-FLU **10b**. However, there was a fluorescence decrease (ca 50%) for TTZ-SGM-FLU **7b** as compared to TTZ-FLU **9b**. In cross-linked AFCs, in both antibody- and dye-dependent manners, the fluorophores could become more exposed to the exchange with a polar aqueous environment that contains quencher species like chlorides and dioxygen [30]. The partial inhibition of their fluorescence emission can therefore occur because of their collisions with quencher species in the buffer. This well-known physical quenching mechanism is independent of the chemical nature of the compound emitting fluorescence. We did not make the experiment in quencher-free medium because it may perturb the AFC stability, both colloidal and conformational. The stronger quenching of fluorescence observed for BODIPY may be additionally explained by its ability to form aggregates (H- or J-type), which is known to occur at high concentrations of BODIPY [31]. Although it cannot be excluded, the latter quenching mechanism seems unlikely in the present study because of (i) the low FAR used, (ii) the absence of fluorescence quenching for BODIPY in the lysine-functionalized AFCs, and (iii) the potential sterical hindrance from linkers, which may not favor the aggregation where molecular units have to be aligned either in a parallel, coplanar, or head-to-tail fashion [32].

### 3.6. Stability of AFCs upon Storage

In order to evaluate AFC stability, we compared the fluorescence emission before and after storage in the dark at 4 °C for 2 months (Figure S9).

All SGM-based AFCs **7a**, **7b**, **8a,** and **8b** were very stable over time in these storage conditions. However, we observed discrepancies in behavior with lysine-conjugated AFCs. Indeed, while TTZ-BDP **9a** and RTX-BDP **10a** were stable over time, TTZ-FLU **9b** and RTX-FLU **10b** showed a fluorescence increase upon storage, which can be explained through an mAb macromolecular structure reorganization, limiting dye exposure to the aqueous buffer, i.e., to the fluorescence quenching by chloride ions [30]. Thus, the lysine-conjugated fluorescein dye became more protected from the aqueous environment after two months of storage. These structural modifications could hamper the reproducibility of assays carried out with lysine-labelled AFCs after storage over long periods of time. Fluorescence emission results demonstrated that cross-linked AFCs were more reliable over time.

### 3.7. Stability of AFCs to pH Variation

Some biological assays, such as FcRn-binding testing (compulsory for the regulatory filing of any mAb-based drug), require slightly acidic conditions (pH 6) [33]. Indeed, this receptor binds mAbs at pH 6 in early endosomes before releasing them in the extracellular environment (neutral in terms of pH) and is responsible for the recycling and, therefore, the long half-life of mAbs. Thus, testing the affinity of the IgG–FcRn complex at pH 6 is crucial during the preclinical development of a new therapeutic mAb [34]. For this reason, we decided to compare the fluorescence emission of our eight AFCs at pH 6 and 7. To that end, we added 1% (v/v) of 2-(*N*-morpholino)ethanesulfonic acid (MES) 100X to the pH 7 solutions in PBS, shifting the pH to 6. We evaluated fluorescence emission without further treatment (Figure S10). 

Upon examining the fluorescence emission of the dye in the AFCs, it appeared that the quantum yield of fluorescein-labelled AFCs **7b**, **8b**, **9b**, and **10b** was more pH-sensitive than the BODIPY-labelled AFCs **7a**, **8a**, **9a**, and **10a**. Indeed, all AFCs containing fluorescein, a pH-sensitive dye [35,36], have lower fluorescence emission (approximately twofold lower) at pH 6 compared to pH 7. BODIPY-functionalized AFCs showed no change in their emission at pH 6. Upon examining the fluorescence emission spectrum of the mAb moiety, it appears that decreasing the pH did not impact the mAb fluorescence emission and, consequently, did not destabilize its folding at lower pH.

## 4. Conclusions

The lack of information on labelling kits and the incomplete characterization of labelled mAbs produced thanks to these induce reproducibility-related issues, aggregation, and, potentially, functional losses. To circumvent these limitations and set up a reproducible labelling method, we generated and compared eight AFCs based on two mAb scaffolds, two fluorophores, and two well-known bioconjugation methods.

We characterized the eight AFCs by mass spectroscopy and studied their fluorescence properties. They exhibited discrepancies between their structure, respective fluorescence emission, stability upon storage, or pH shift. SGM-based AFCs were more homogeneous than their lysine-labelled counterparts. We observed a quenching of fluorescence emission for SGM linkers, which was dye- and antibody-dependent. However, we observed that SGM-based AFCs were more stable upon storage and equally stable to pH shifts when compared to their lysine-labelled counterparts.

The better stability of SGM linkers could be of interest for future in vitro applications. We demonstrated that a labelling experiment does not proceed similarly according to the antibody of interest, and resulting AFCs exhibit discrepancies in a dye-, antibody-, and labeling-method-dependent manner. Furthermore, with the replacement of green-emitting fluorophores by near-infrared dyes, this methodology could also be applied to carry out in vivo experiments.

## Figures and Tables

**Table 1:** Antibody–fluorophore conjugates (AFCs) obtained from interchain cysteine cross-linking or lysine bioconjugation technologies onto trastuzumab or rituximab with BODIPY or fluorescein dyes.

Antibody	Compound Used for Bioconjugation	AFC
Trastuzumab	BODIPY-SGM **6a**	TTZ-SGM-BDP **7a**
Trastuzumab	Fluorescein-SGM **6b**	TTZ-SGM-FLU **7b**
Rituximab	BODIPY-SGM **6a**	RTX-SGM-BDP **8a**
Rituximab	Fluorescein-SGM **6b**	RTX-SGM-FLU **8b**
Trastuzumab	BODIPY-NHS	TTZ-BDP **9a**
Trastuzumab	Fluorescein-NHS	TTZ-FLU **9b**
Rituximab	BODIPY-NHS	RTX-BDP **10a**
Rituximab	Fluorescein-NHS	RTX-FLU **10b**

## Supplementary Materials

The following are available online at https://www.mdpi.com/1424-8247/12/4/176/s1: synthesis of starting compound 5, NMR spectra, MS spectra, RAW MS spectra, TIC, UV-visible, ELISA and cytometry data. Scheme S1: Synthesis of compounds 1, 2, 3, 4 and 5. Table S1: Optimized equivalents for lysine conjugation. Table S2 and Table S3: Molecular weights of AFCs expected and observed by MS. Table S4. Statistical analysis on CD20 binding. Figure S1: Deconvoluted spectra of AFCs and native trastuzumab. Deglycosylation was performed using PNGase F on lysines samples diluted to 1 mg/mL. Samples were incubated 6 h at 37 °C prior to MS analysis. Figure S2: Deconvoluted spectra of AFCs and native rituximab. Deglycosylation was performed using PNGase F on lysines samples diluted to 1 mg/mL. Samples were incubated 6 h at 37 °C prior to MS analysis. Figure S3: Raw mass spectra of native and deglycosylated mAbs, AFCs and associated TIC Native RTX. Figure S4. HER2 affinities of AFCs TTZ-SGM-FLU **7b** (blue curve) and TTZ-FLU **9b** (orange curve) by ELISAs. Figure S5. Binding of AFCs RTX-SGM-FLU **8b** (blue curve) and RTX-FLU **10b** (orange curve) to CD20 by flow cytometry. Figure S6. Models representing rituximab (left) and trastuzumab (right), from PDB structures 2OSL and 4HKZ respectively. Heavy (blue) and light (red) chains are represented as cartoons, and lysines are visualized as cyan surfaces. In the V-domains, trastuzumab contains 9 lysine residues (4 in VH and 5 in VL), among which one is contained inside CDRH1 and 2 others in the heavy chain framework are close to the paratope. For rituximab, the V-domains include 12 lysine residues among which 6, all inside the heavy chain, are very close to the paratope. Figure S7. Flow cytometry of AFCs RTX-SGM-FLU 8b and RTX-FLU 10b at pH 7 and pH 6, n = 3. UV-visible absorption of AFCs. Sample concentration was adjusted to 500 µg/mL of protein. Figure S8. UV-visible absorption of AFCs. Sample concentration was adjusted to 500 µg/mL of protein. Figure S9. Fluorescence emission of fresh (orange curves) and stored in the dark at 4 °C (blue curves) AFCs based on rituximab after normalisation. Excitation wavelength: 275 nm. For easier comparison, the sample concentration was adjusted to 50 µg/mL of protein. Figure S10. Comparison of fluorescence emission of AFCs based on rituximab at pH 7 (blue curves) and 6 (orange curves) after normalisation. Excitation wavelength: 275 nm. For easier comparison, the sample concentration was adjusted to 50 µg/mL of protein.
