# Supplementary material for: In Vitro Characterization and Stability Profiles of Antibody–Fluorophore Conjugates Derived from Interchain Cysteine Cross-Linking or Lysine Bioconjugation
Source: Pharmaceuticals (Basel). 2019 Dec 2;12(4):176. doi: 10.3390/ph12040176 (PMC6958397; doi:10.3390/ph12040176)
Supplement: Supplementary file 1 [file pharmaceuticals-12-00176-s001.pdf]

Supporting Information

# *In vitro* characterization and stability profiles of antibody-fluorophore conjugates generated through cross-linking or lysine bioconjugation.

**Camille Martin [1], Guillaume Brachet [2,3], Cyril Colas [4,5], Emilie Allard-Vannier [6], Claire Kizlik-Masson [3], Clara Esnault [1], Renaud Respaud [7], Caroline Denevault-Sabourin [1], Igor Chourpa [6], Valérie Gouilleux-Gruart [2,3], Marie-Claude Viaud-Massuard [1], and Nicolas Joubert [1,*]**

[1] GICC EA7501, Equipe IMT, Université de Tours, UFR des Sciences Pharmaceutiques, 31 Avenue Monge, 37200 Tours, France.
[2] Laboratoire d'immunologie, CHRU Tours, 10 Bd Tonnelé, 37000 Tours, France.
[3] GICC EA7501, Equipe FRAME, Université de Tours, 10 Bd Tonnelé, 37000 Tours, France.
[4] ICOA UMR 7311 CNRS, Université d'Orléans, Pôle de Chimie, Rue de Chartres, BP 6759, 45067 Orléans Cedex 2, France.
[5] CBM, UPR4301 CNRS, Rue Charles Sadron, 45071 Orléans, France.
[6] NMNS EA 6295, 31 Avenue Monge, 37200 Tours, France.
[7] INSERM U1100, Université de Tours, 10 Bd Tonnelé, 37000 Tours, France.
* Correspondence: nicolas.joubert@univ-tours.fr

**Table of content**

Synthesis of compounds **1**, **2**, **3**, **4** and **5** ..... S2
NMR spectra of new compounds ..... S4
Optimized equivalents for lysine conjugation ..... S12
Deconvoluted spectra of AFCs and native mAbs ..... S12
Molecular weights of AFCs expected and observed by MS ..... S14
Raw mass spectra of native and deglycosylated mAbs, AFCs and associated TIC ..... S15
HER2 binding of AFCs TTZ-SGM-FLU **7b** and TTZ-FLU **9b** ..... S28
CD20 binding of AFCs RTX-SGM-FLU **8b**, RTX-FLU **10b** at pH7 and pH6, n = 3 ..... S28
Statistical analysis on CD20 binding ..... S29
Models representing paratopes of rituximab and trastuzumab ..... S29
UV-visible absorption of AFCs ..... S30
Stability of AFCs upon storage ..... S31
Stability of AFCs to pH variation ..... S32

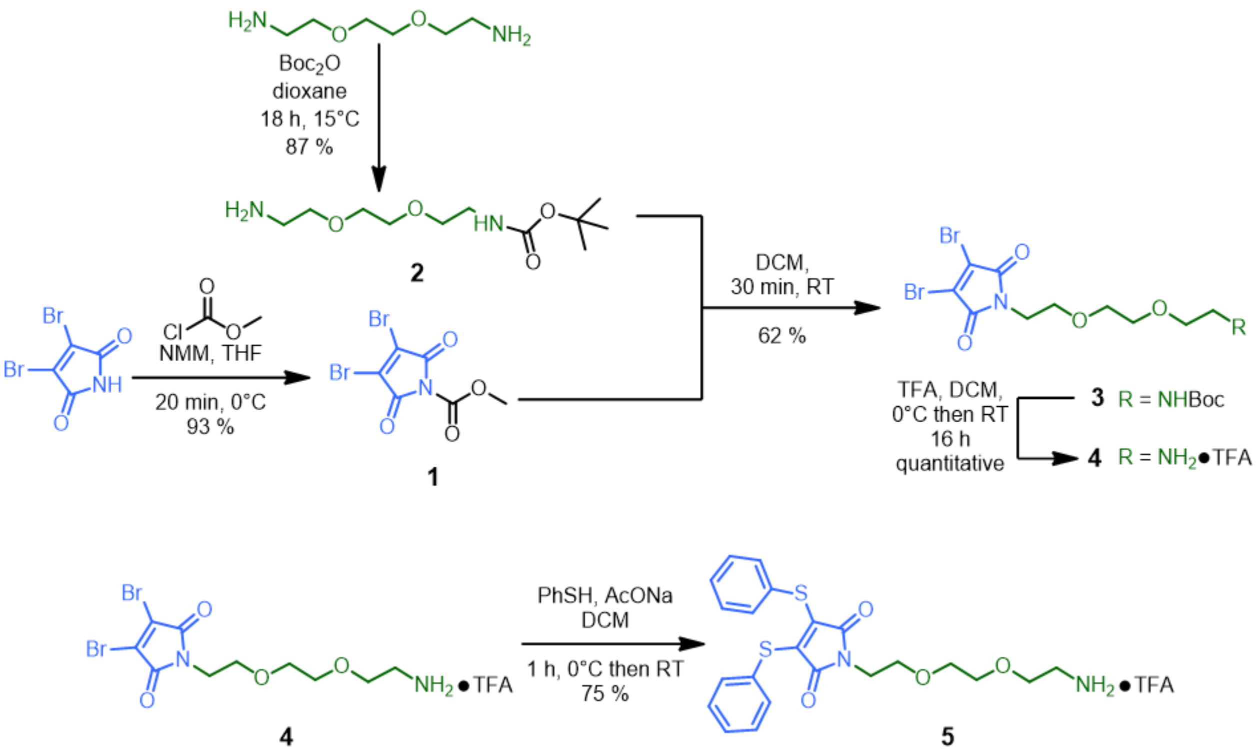


**Scheme S1.** Synthesis of compounds **1, 2, 3, 4 and 5**

*2,3-Dibromo-*N*-methoxycarbonylmaleimide* **1**

2,3-Dibromomaleimide (2.82 g, 11.065 mmol) was dissolved in dry tetrahydrofuran (99 mL) and cooled down to 0 °C. *N*-Methylmorpholine (1.220 mL, 11.065 mmol) and methylchloroformate (854 µL, 11.065 mmol) were added and stirred at 0 °C for 20 min under inert atmosphere. Dichloromethane was added (120 mL) and the organic phase was washed with water (3×40 mL), dried over magnesium sulphate ($MgSO_4$) and concentrated under reduced pressure to give **1** as a purple solid (3.21 g, 93 %). $^1$H NMR (300 MHz, $CDCl_3$) δ (ppm) 4.00 (s, 3H, $CH_3$). $^{13}$C NMR (75 MHz, $CDCl_3$) δ (ppm) 159.3 (×2), 147.0, 131.5 (×2), 54.8.[1]

*1-(*t*-Butyloxycarbonyl-amino)-3,6-dioxa-8-octaneamine* **2**

1,8-Diamino-3,6-dioxaoctane (1.058 mL, 7.254 mmol) was dissolved in dioxane (4 mL) and cooled down to 10-15 °C, di-*t*-butyl pyrocarbonate (264 mg, 1.209 mmol) in dioxane (4 mL) was added slowly at 10-15 °C and stirred at 15 °C overnight. Dioxane was removed under reduced pressure and water was added. The aqueous residue was extracted with ethyl acetate (5×50 mL), combined organic phases were washed with brine (5×50 mL), dried over $MgSO_4$ and concentrated under reduced pressure to give **2** (261 mg, 87%) as a colourless oil. $^1$H NMR (300 MHz, $CDCl_3$) δ (ppm) 5.18 (broad s, 1H, NH(CO)), 3.62-3.53 (m, 8H, $CH_2$-O), 3.53-3.31 (m, 2H, $CH_2$-N), 2.90 (t, $J$ = 4.9 Hz, 2H, $CH_2$-NH(CO)), 1.44 (s, 9H, $CH_3$).[2]

*3,4-Dibromomaleimide-*N*-8-(*t*-Butyloxycarbonyl-amino)-3,6-dioxa-octane* **3**

**1** (500 mg, 1.028 mmol) was dissolved in dry dichloromethane (6.6 mL), a solution of freshly prepared **2** (397 mg, 1.028 mmol) in dry dichloromethane (6.6 mL) was added and it was stirred under argon at room temperature for 30 min. Water was added and the organic phase was

washed with water (3×15 mL), dried $MgSO_4$ and solvent was removed by evaporation. The crude mixture was purified by flash chromatography ($SiO_2$, dichloromethane/methanol 99:1) to give **3** (485 mg, 62%) as a pale beige solid. $^1$H NMR (300 MHz, $CDCl_3$) δ (ppm) 4.98 (broad s, 1H, NH(CO)), 3.83 (t, $J$ = 5.4 Hz, 2H $CH_2$), 3.67 (t, $J$ = 5.4 Hz, 2H, $CH_2$-O), 3.62-3.58 (m, 2H, $CH_2$-O), 3.58-3.53 (m, 2H, $CH_2$-O), 3.50 (t, $J$ = 5.1 Hz, 2H, $CH_2$-O), 3.33-3.26 (m, 2H, $CH_2$-NH(CO)), 1.44 (s, 9H, $CH_3$). $^{13}$C NMR (75 MHz, $CDCl_3$) δ (ppm) 163.9 (×2), 156.0, 129.5 (×2), 77.2, 70.3, 70.2, 70.0, 67.6, 38.8 (×2), 28.4 (x3). HRAM (ESI): *m/z* calc.= 484.9917, found 484.9911.

*3,4-Dibromomaleimide*-N-*8-amino-3,6-dioxaoctane* **4**

**3** (284 mg, 0.584 mmol) was dissolved in dry dichloromethane ($CH_2Cl_2$) (6.3 mL) and cooled down to 0 °C. Trifluoroacetic acid (TFA) (626 µL, 10% solution in $CH_2Cl_2$) was added at 0 °C and it was stirred at room temperature (rt) overnight. The crude was concentrated under reduced pressure and TFA was co-evaporated with $CH_2Cl_2$ (3×30 mL) to give the trifluoroacetic salt of **4** (295 mg, 100%) as a white solid. $^1$H NMR (300 MHz, $CDCl_3$) δ (ppm) 7.74 (broad s, 3H, $NH_3$), 3.83 (t, $J$ = 9.9 Hz, 2H, $CH_2$), 3.74 (t, $J$ = 9.7 Hz, 2H, $CH_2$-O), 3.67-3.58 (m, 6H, $CH_2$-O), 3.30-3.21 (m, 2H, $CH_2$-N). $^{13}$C NMR (75 MHz, $CDCl_3$) δ (ppm) 164.5 (×2), 159.9, 129.7 (×2), 77.4, 70.1, 69.9, 68.3, 66.3, 40.2, 39.2. HRAM (ESI): *m/z* calc.= 384.9393, found 384.9392.

*3,4-Dithiophenoylmaleimide*-N-*8-amino-3,6-dioxaoctane* **5**

A solution of the trifluoroacetic salt of **4** (52 mg, 0.103 mmol) in dry $CH_2Cl_2$ (1 mL) was added dropwise to an ice-cold stirring solution of sodium acetate (27 mg, 0.309 mmol) and thiophenol (31 µL, 0.297 mmol) in dry $CH_2Cl_2$ (1 mL). The solution turned yellow and was stirred at rt for 1 h. It was cooled down to 0 °C and treated with HCl 1N dropwise. After $CH_2Cl_2$ extractions (3×10 mL), organic phases were combined, dried over $MgSO_4$ and concentrated under reduced pressure to give an orange oil. It was taken up in a minimum amount of $CH_2Cl_2$ and put on a cake of silica. It was washed with cyclohexane to remove the excess of thiophenol and then used as a solid deposit for purification by flash chromatography ($SiO_2$, $CH_2Cl_2$/MeOH 97:3) to give the trifluoroacetic salt of **5** (43 mg, 75%) as an orange oil. $^1$H NMR (300 MHz, $CDCl_3$) δ (ppm) 8.12 (s broad, 3H, $NH_3$), 7.26-7.16 (m, 10H, Ph), 3.80-3.48 (m, 10H, $CH_2$, $CH_2$-O), 3.20-3.09 (m, 2H, $CH_2$-N). $^{13}$C NMR (75 MHz, $CDCl_3$) δ 167.3 (×2), 135.8, 132.0 (×4), 129.2 (×4), 129.0 (×4), 128.6 (×2), 77.4, 70.1, 69.9, 68.2, 66.4, 40.2, 38.5. $^{19}$F (282 MHz, $CDCl_3$) δ (ppm) -75. HRAM (ESI): *m/z* calc.= 445.1250, found 445.1250.

Compound 5 – 1H NMR

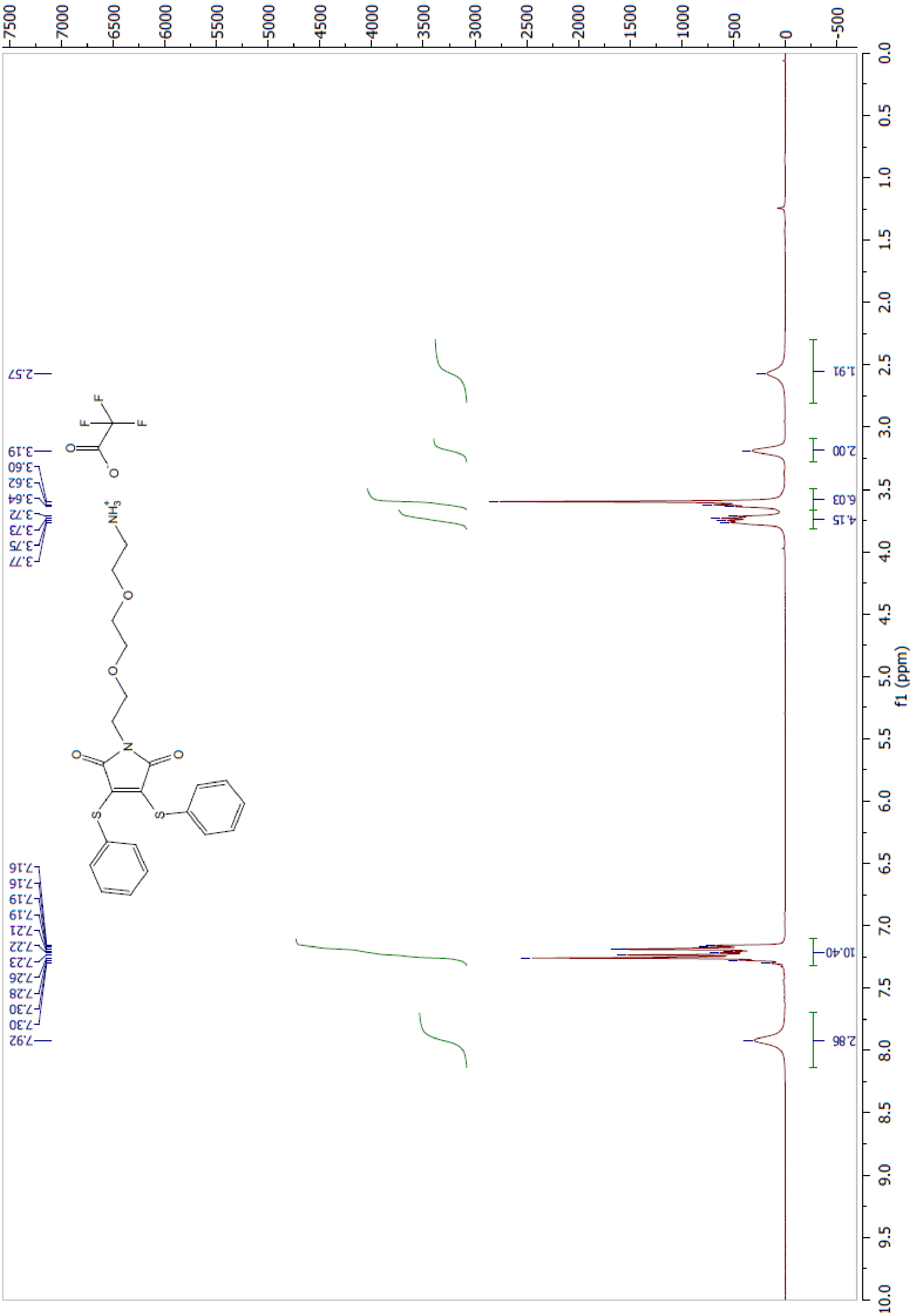

Compound 5 – 13C NMR

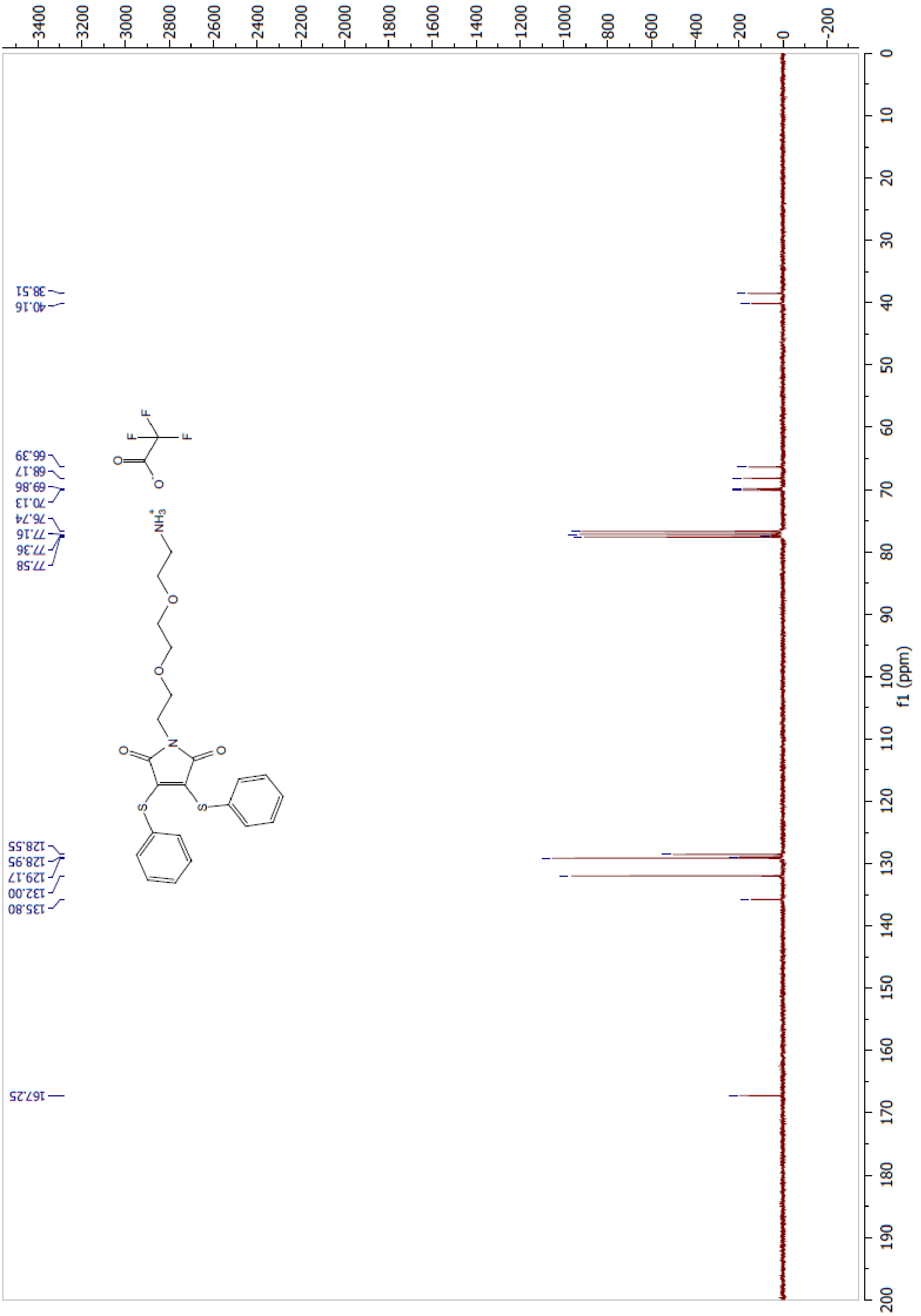

Compound 5 – 19F NMR

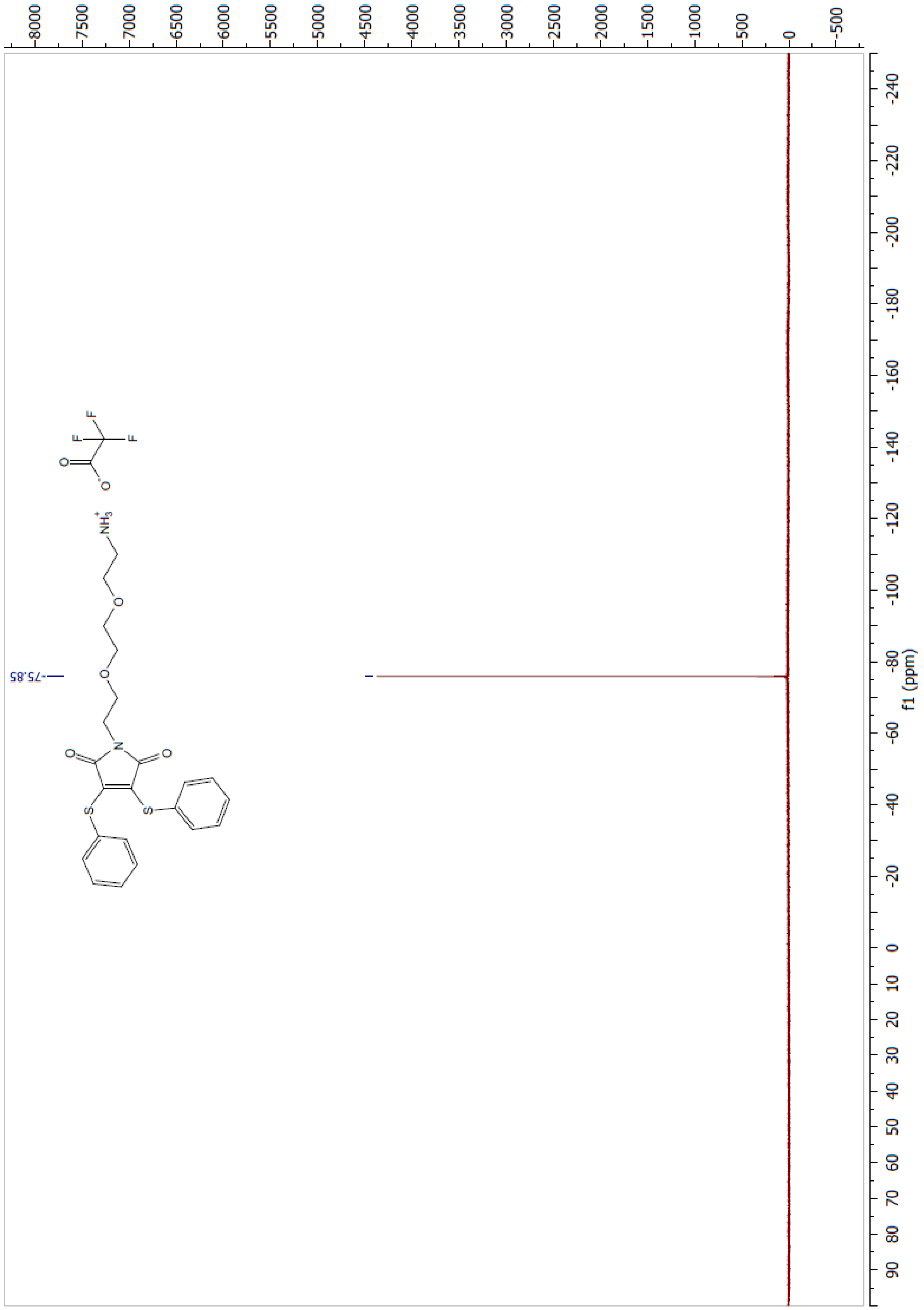

Compound 6a – 1H NMR

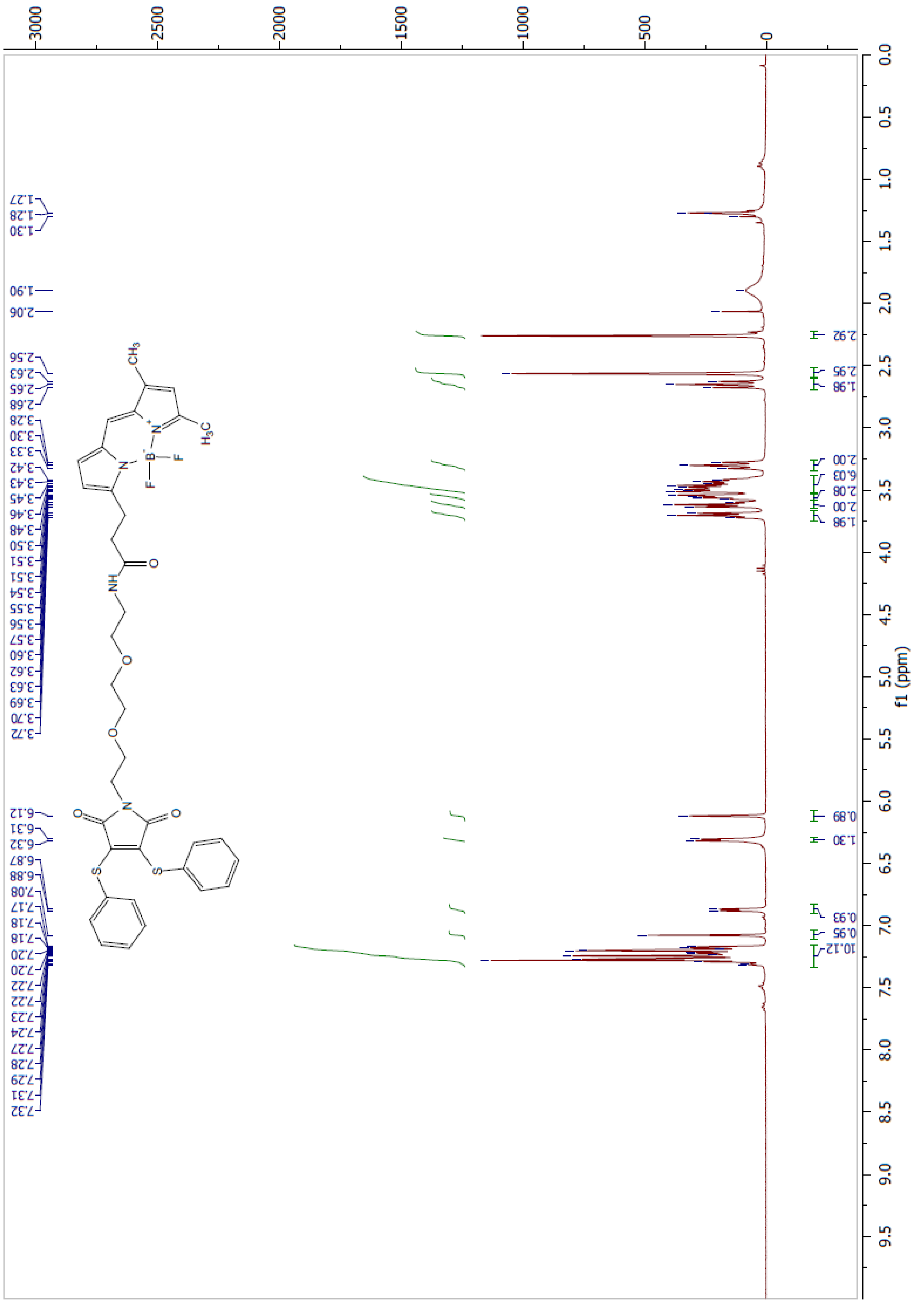

Compound 6a – 13C NMR

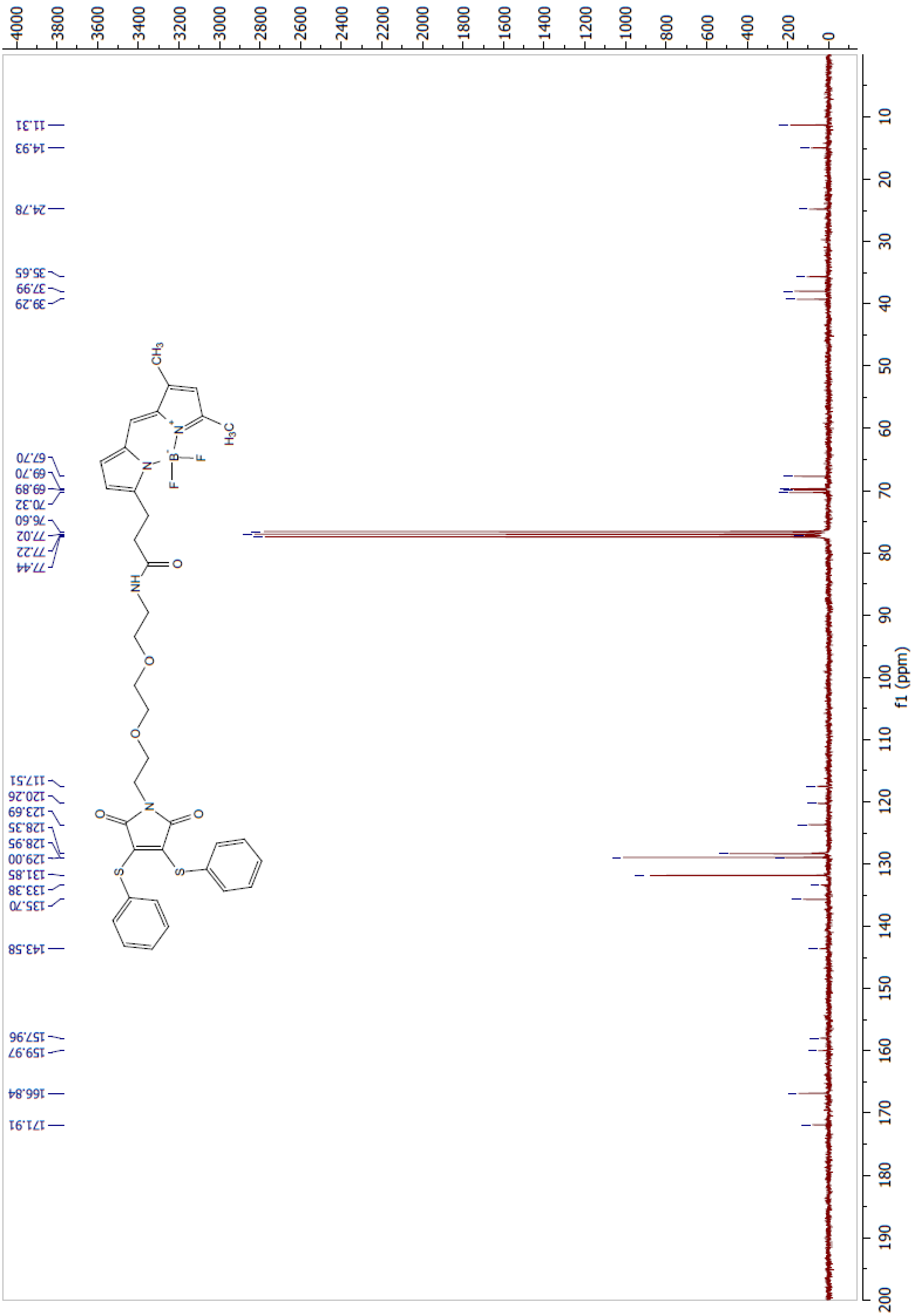

Compound 6a – 19F NMR

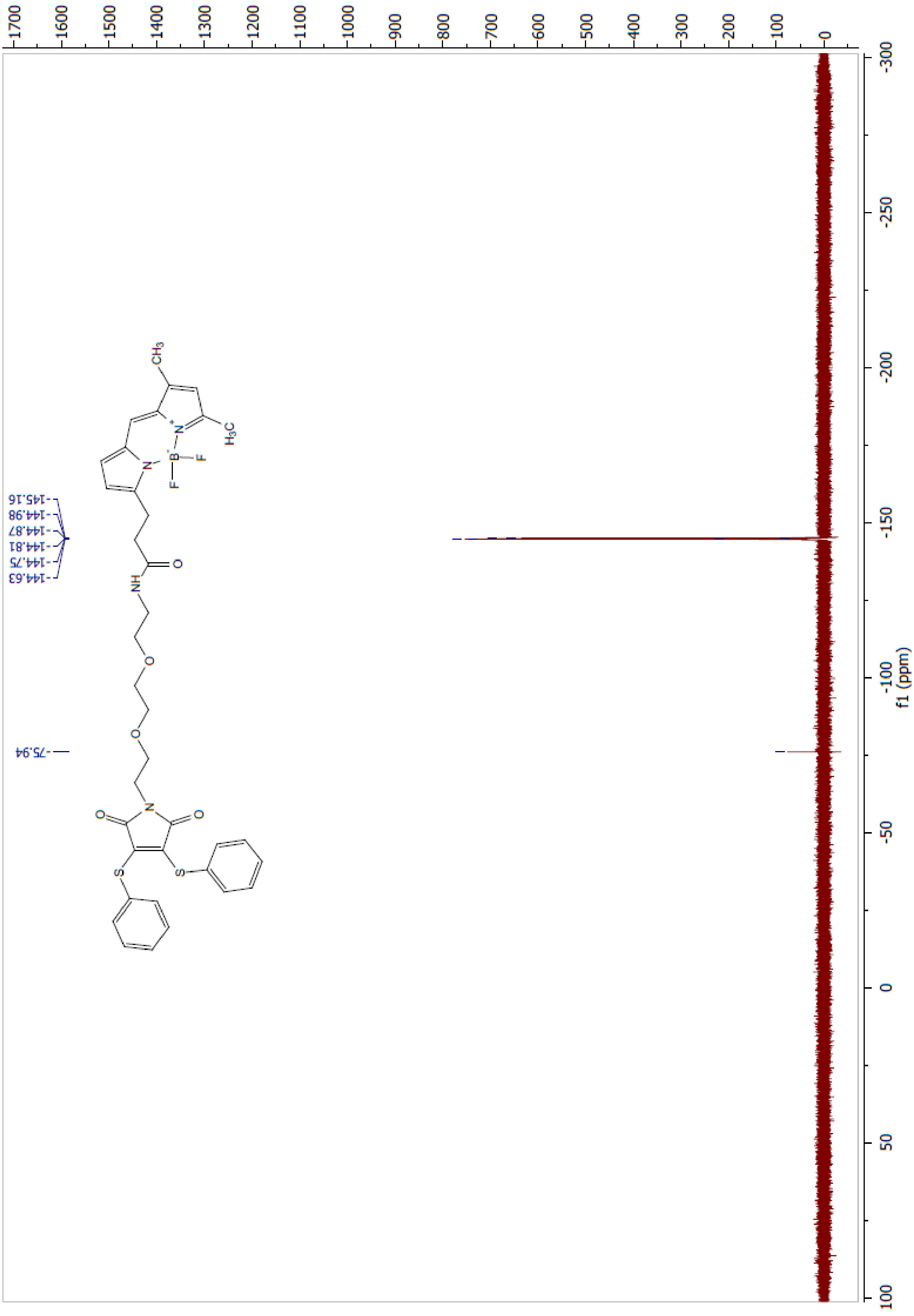

Compound 6b – 1H NMR

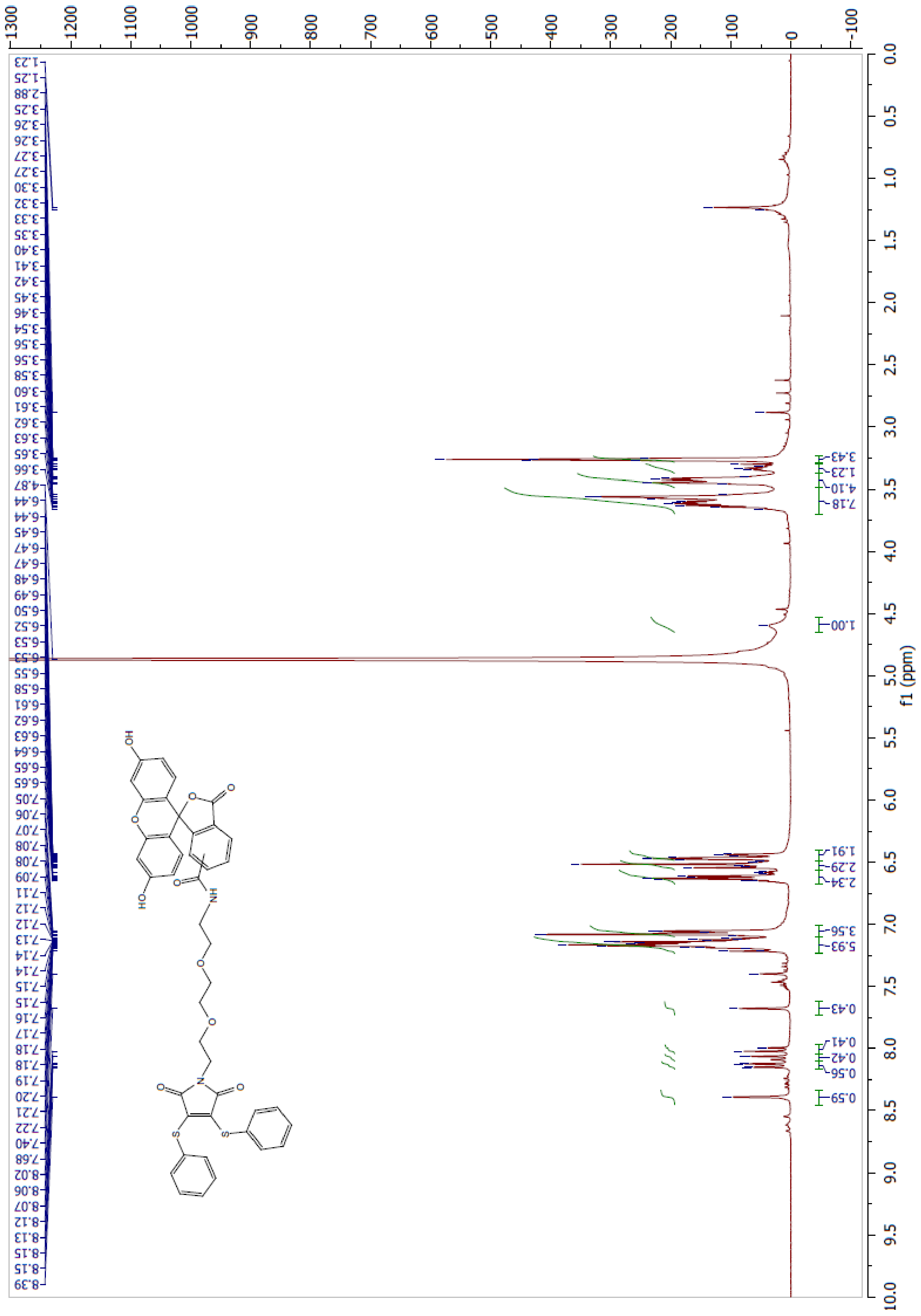

Compound 6b – 13C NMR

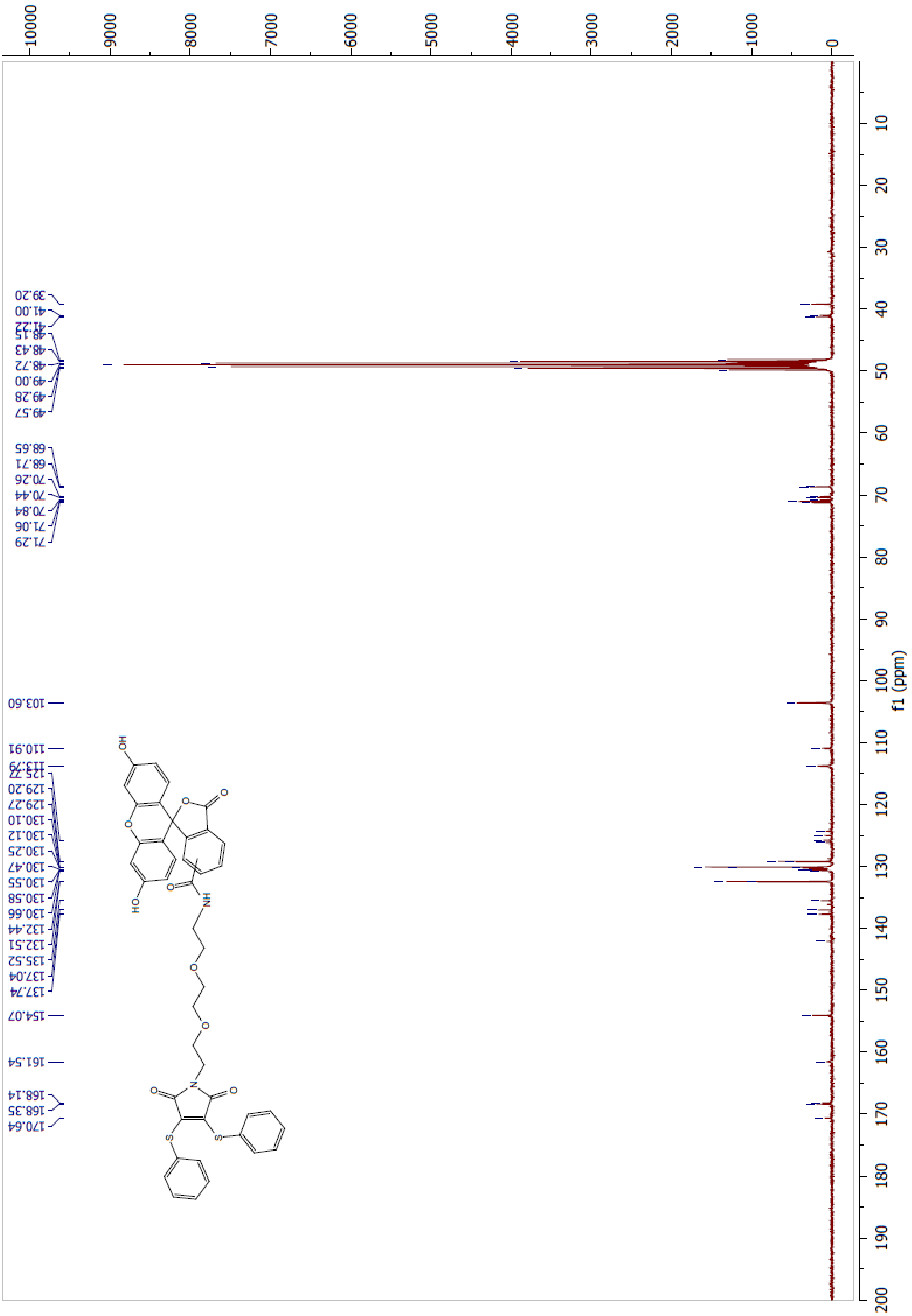

**Table S1.** Optimized equivalents for lysine conjugation

| mAb | Fluorophore | eq | Volume of 0.01M (µL) |
|---|---|---|---|
| TTZ | BDP | 3.3 | 4.0 |
| TTZ | FLU | 10 | 12 |
| RTX | BDP | 2.7 | 3.2 |
| RTX | FLU | 10 | 12 |

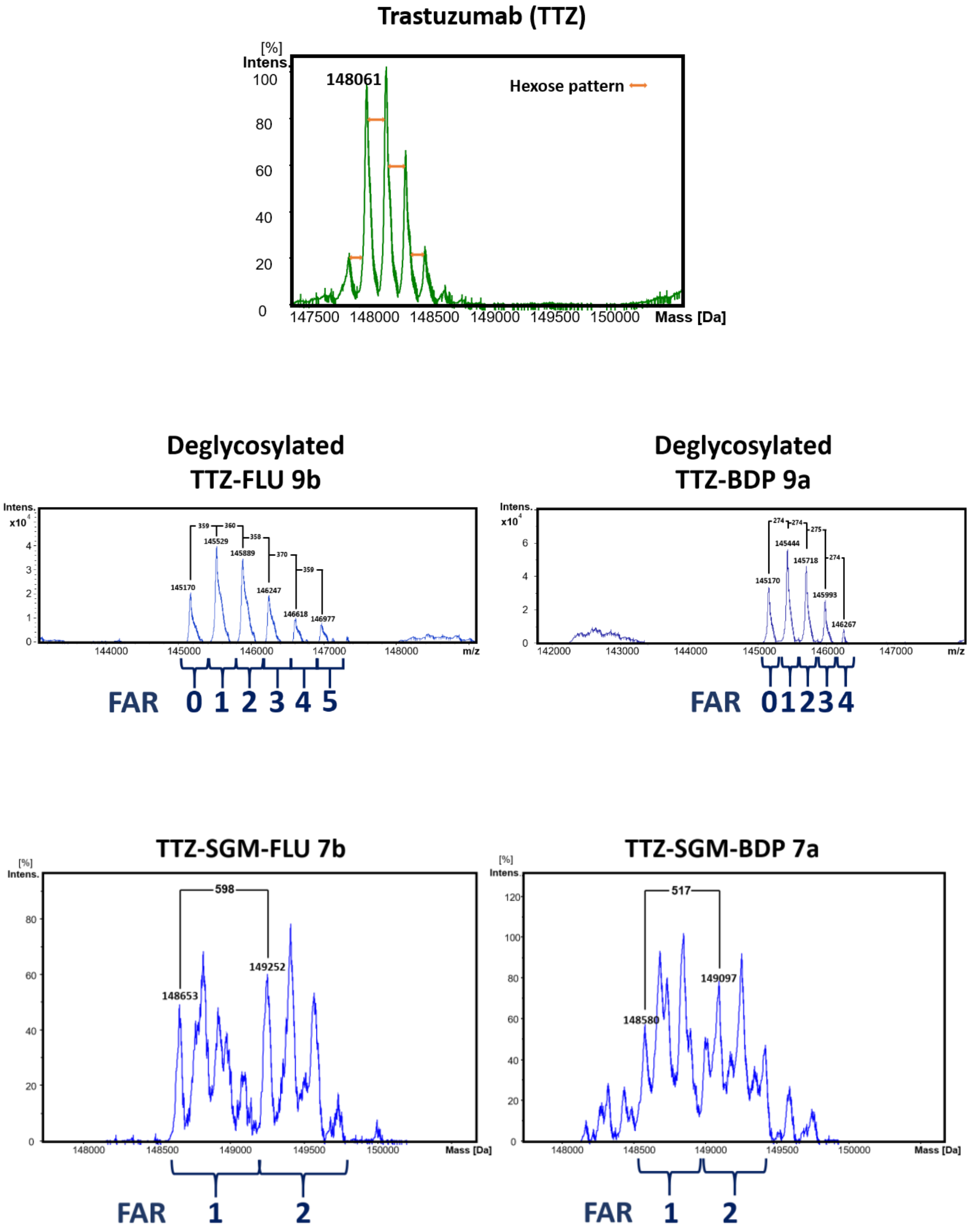


**Figure S1.** Deconvoluted spectra of AFCs and native trastuzumab. Deglycosylation was performed using PNGase F on lysines samples diluted to 1 mg/mL. Samples were incubated 6 h at 37 °C prior to MS analysis.

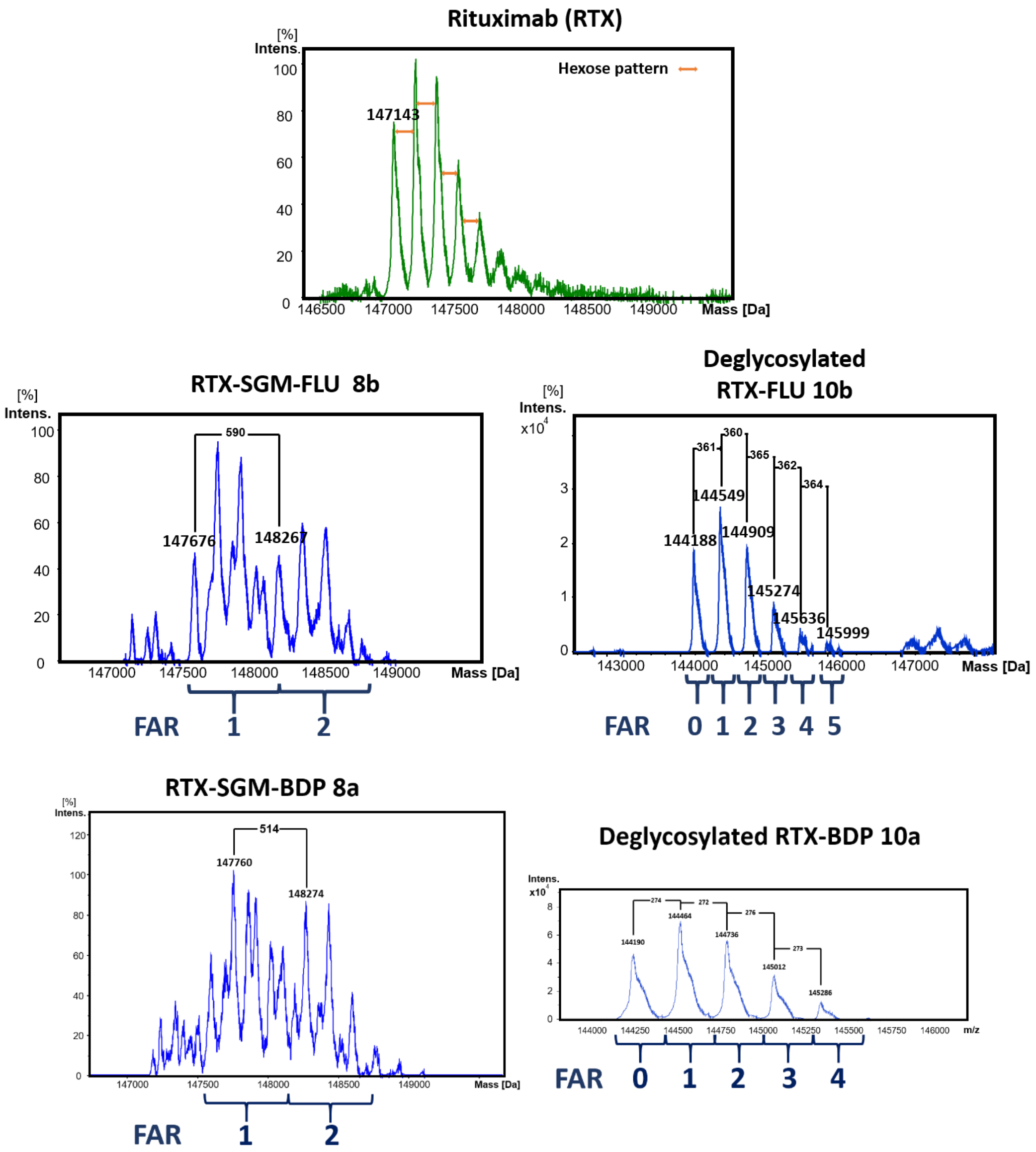


**Figure S2.** Deconvoluted spectra of AFCs and native rituximab. Deglycosylation was performed using PNGase F on lysines samples diluted to 1 mg/mL. Samples were incubated 6 h at 37 °C prior to MS analysis.

Mass spectrometry analysis was used to determinate FAR distribution of all AFCs. We used an electrospray ionisation (ESI) source, producing multi-charged ions. Classical glycosylation profiles of native antibodies were observed. For labelled mAbs, average FAR was calculated from signal integration of deconvoluted mass spectra, either on glycosylated or deglycosylated proteins. The small mass increment resulting for lysine conjugation required deglycosylation before analysis to avoid signal overlap between glycosylation and conjugation and to identify each FAR species unambiguously. While mass increment resulting from cross-linking was higher and sufficiently important to allow discrepancies between different FAR species. This is why, for cross-linking labelling technology, we chose to analyse the unmodified glycosylated AFC, while we deglycosylated the AFCs resulting from lysine labelling.

Second generation maleimide (SGM) are heterocycle known in the antibody-drug conjugate (ADC) field of research. For bioconjugation using SGM (dithiophenylmaleimide or dibromomaleimide) you can see the published work of our group (*Org. Biomol. Chem.*, **2018**, *16*, 1882–1889; *Bioconjugate Chem*. **2018**, *29*, 3516–3521; patent WO2015004400), from Jackson *et al.* (Igenica; *Mol. Pharmaceutics* **2015**, *12*, 3986−3998) or from Baker *et al.* (UCL; *Org. Biomol. Chem.*, **2014**, *12*, 7261-7269, etc).

Classical glycosylation profiles of native antibodies were observed. For labelled mAbs, average FAR was calculated from signal integration of deconvoluted mass spectra, either on glycosylated or deglycosylated proteins. The small mass increment resulting for lysine conjugation required deglycosylation before analysis to identify each FAR species. While mass increment resulting from cross-linking was higher and sufficiently important to allow discrepancies between different FAR species. This is why, for cross-linking labelling technology, we chose to analyze the unmodified glycosylated AFC, while we deglycosylated the AFCs resulting from lysine labelling. SGM is able to cross-link previously reduced disulfide bridges, as demonstrated in these several papers. Experimentally, it is very simple to verify: for example for the linker **6a** (BODIPY-SGM), the mass increment when conjugated on antibody is 520 Da per dye conjugated, which correspond to the mass of the linker minus two SPh moieties (2 x 109). One SPh is removed from the linker only after bioconjugation of a sulfydryl from a cysteine. Therefore losing two SPh means that the linker is fixed on two cysteine residues (see TTZ-SGM-BDP **7a** in Figure S2 for an example). The average FAR (or DAR) calculation method has been validated in our previous papers (quoted above).

**Table S2** and **Table S3.** Molecular weights of AFCs expected and observed by MS

| AFC | MW FAR0 | | MW FAR1 | | MW FAR2 | | MW FAR3 | | MW FAR4 | | MW FAR5 | |
|---|---|---|---|---|---|---|---|---|---|---|---|---|
| | Exp. | Obs. | Exp. | Obs. | Exp. | Obs. | Exp. | Obs. | Exp. | Obs. | Exp. | Obs. |
| TTZ-BDP **9a** | 145170 | 145170 | 145444 | 145444 | 145718 | 145718 | 145992 | 145993 | 146266 | 146267 | 146540 | - |
| TTZ-FLU **9b** | 145170 | 145170 | 145525 | 145529 | 145880 | 145889 | 146235 | 146247 | 146590 | 146618 | 146945 | 146977 |
| RTX-BDP **10a** | 144187 | 144190 | 144461 | 144464 | 144735 | 144736 | 145009 | 145012 | 145283 | 145286 | 145557 | - |
| RTX-FLU **10b** | 144187 | 144188 | 144542 | 144549 | 144897 | 144909 | 145252 | 145274 | 145607 | 145636 | 145962 | 145999 |

| AFC | MW FAR1 | | MW FAR2 | |
|---|---|---|---|---|
| | Exp. | Obs. | Exp. | Obs. |
| TTZ-SGM-BDP **7a** | 148650 | 148653 | 149240 | 149252 |
| TTZ-SGM-FLU **7b** | 148575 | 148580 | 149090 | 149097 |
| RTX-SGM-BDP **8a** | 147732 | 147676 | 148322 | 148267 |
| RTX-SGM-FLU **8b** | 147657 | 147660 | 148172 | 148274 |

To assess the fluorophore-to-antibody ratio (FAR), we looked at the mass increase between the different species in solution and compared expected (Exp.) and observed (Obs.) masses. Arbitrarily, for samples **7** and **8**, we chose to focus on the first intense glycosylation of antibodies as the value for FAR0.

**Table 1.** AFCs obtained from disulfide cross-linking or lysine bioconjugation technologies onto trastuzumab or rituximab with BODIPY or fluorescein dyes.

| Antibody | Compound used for bioconjugation | AFC |
|---|---|---|
| Trastuzumab | BODIPY-SGM **6a** | TTZ-SGM-BDP **7a** |
| Trastuzumab | Fluorescein-SGM **6b** | TTZ-SGM-FLU **7b** |
| Rituximab | BODIPY-SGM **6a** | RTX-SGM-BDP **8a** |
| Rituximab | Fluorescein-SGM **6b** | RTX-SGM-FLU **8b** |
| Trastuzumab | BODIPY-NHS | TTZ-BDP **9a** |
| Trastuzumab | Fluorescein-NHS | TTZ-FLU **9b** |
| Rituximab | BODIPY-NHS | RTX-BDP **10a** |
| Rituximab | Fluorescein-NHS | RTX-FLU **10b** |

**Figure S3.** Raw mass spectra of native and deglycosylated mAbs, AFCs and associated TIC

Native RTX

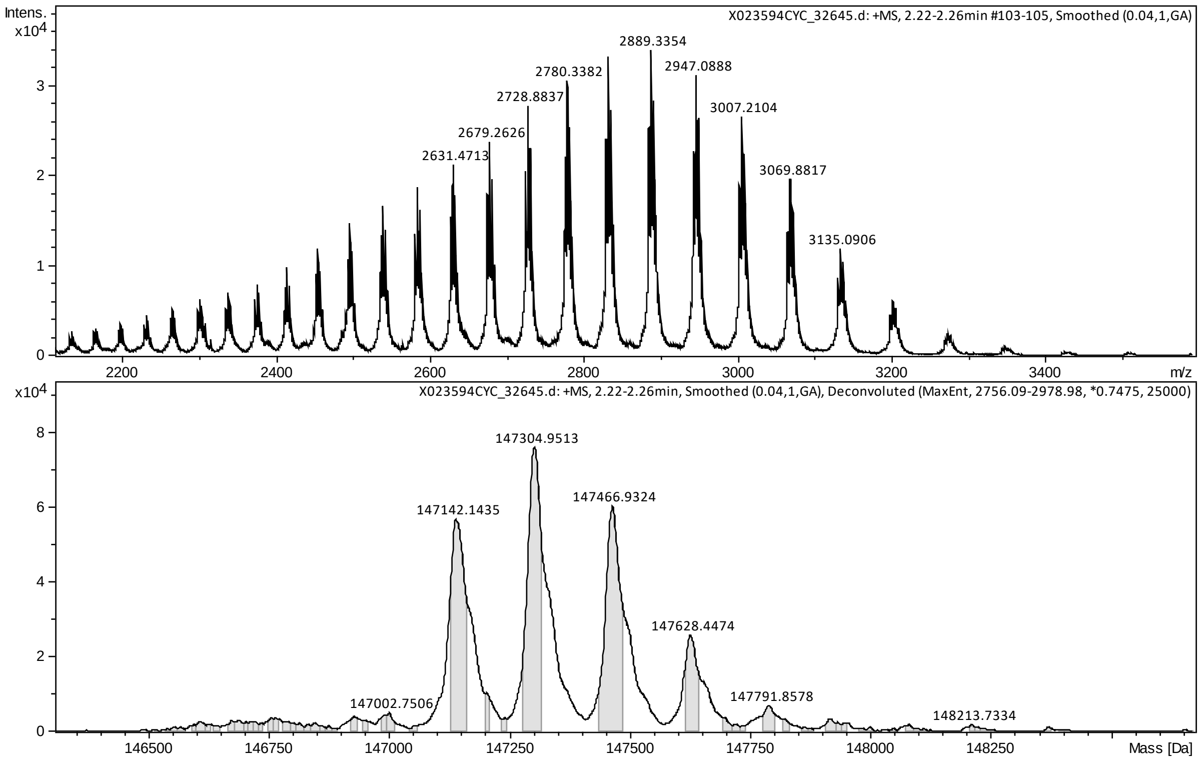


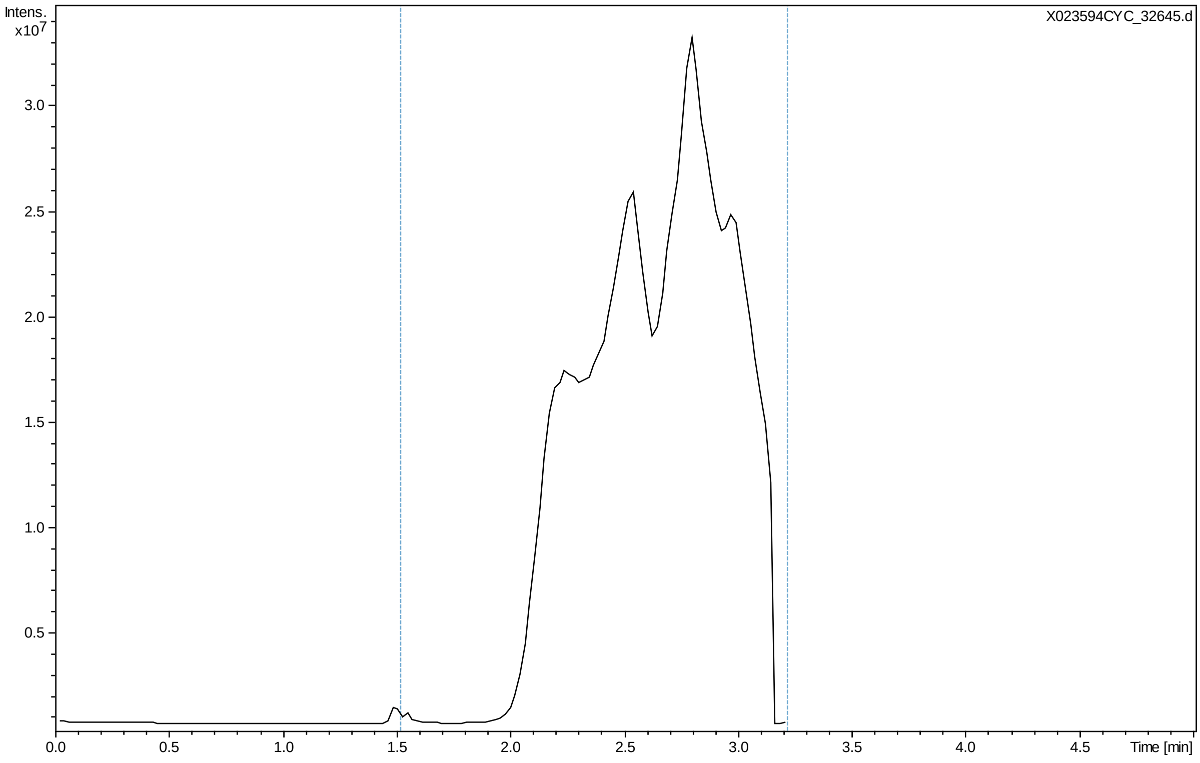

Deglycosylated RTX

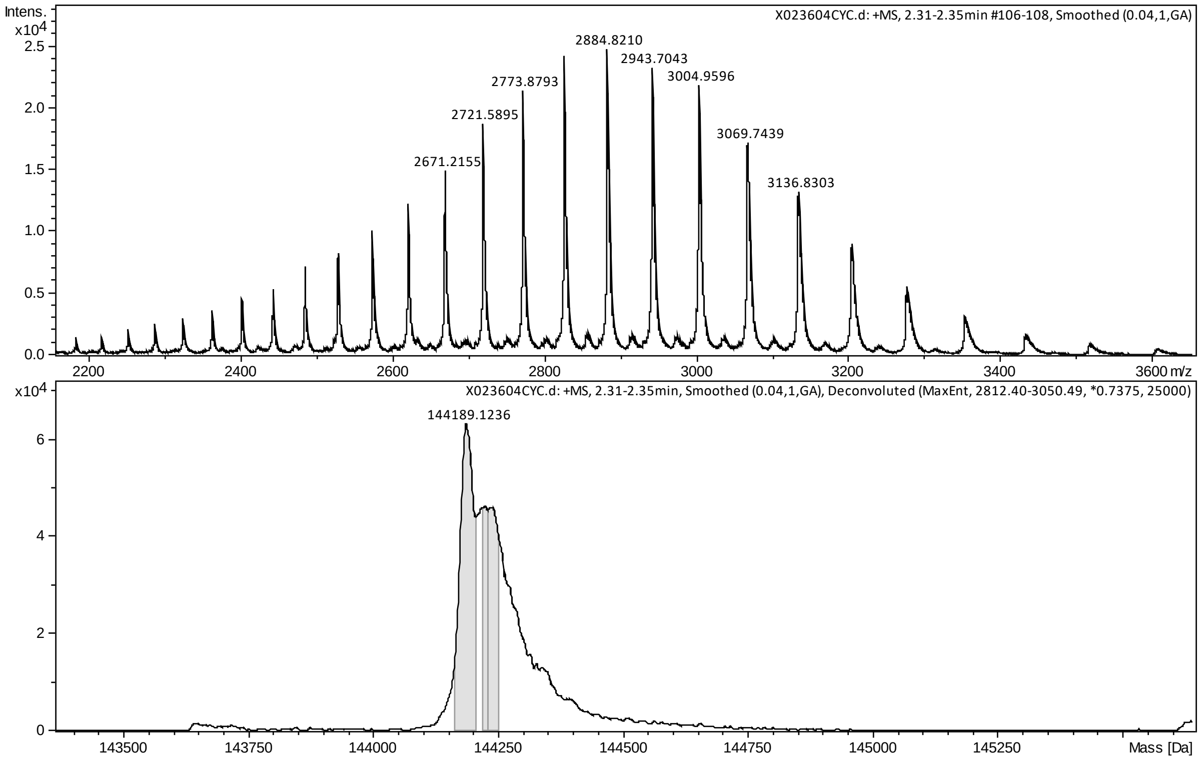


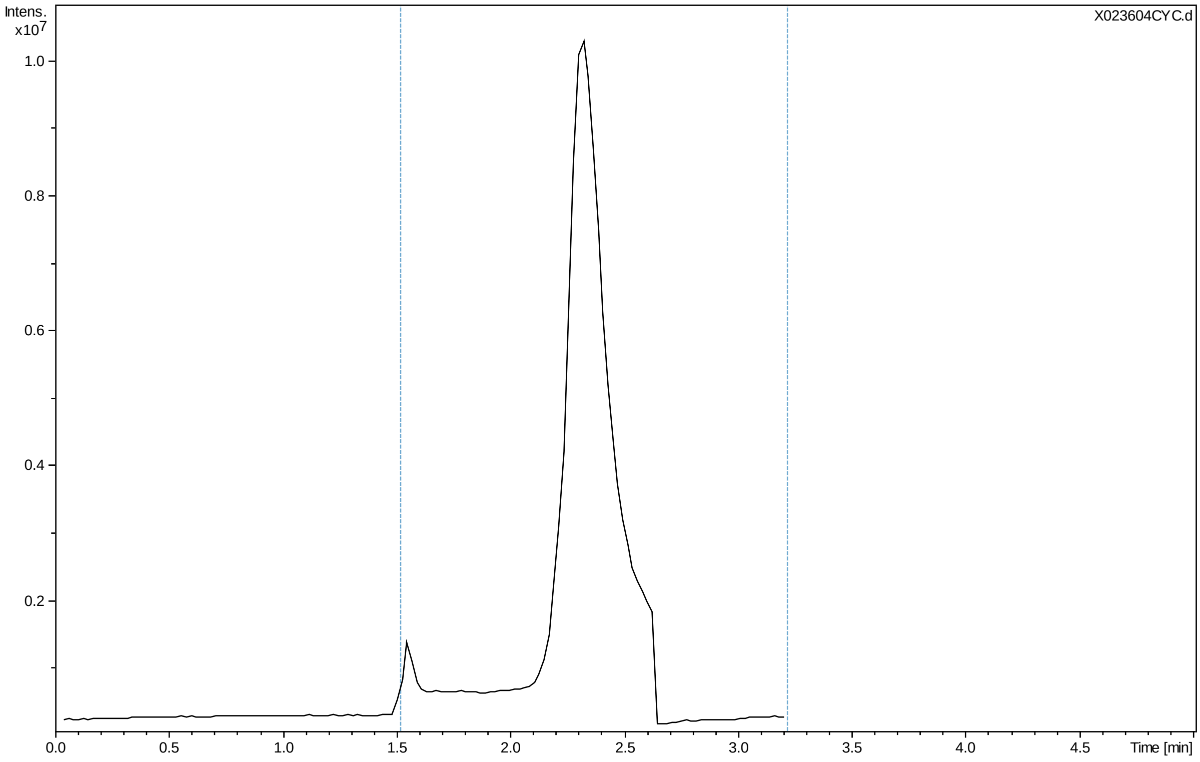

Native TTZ

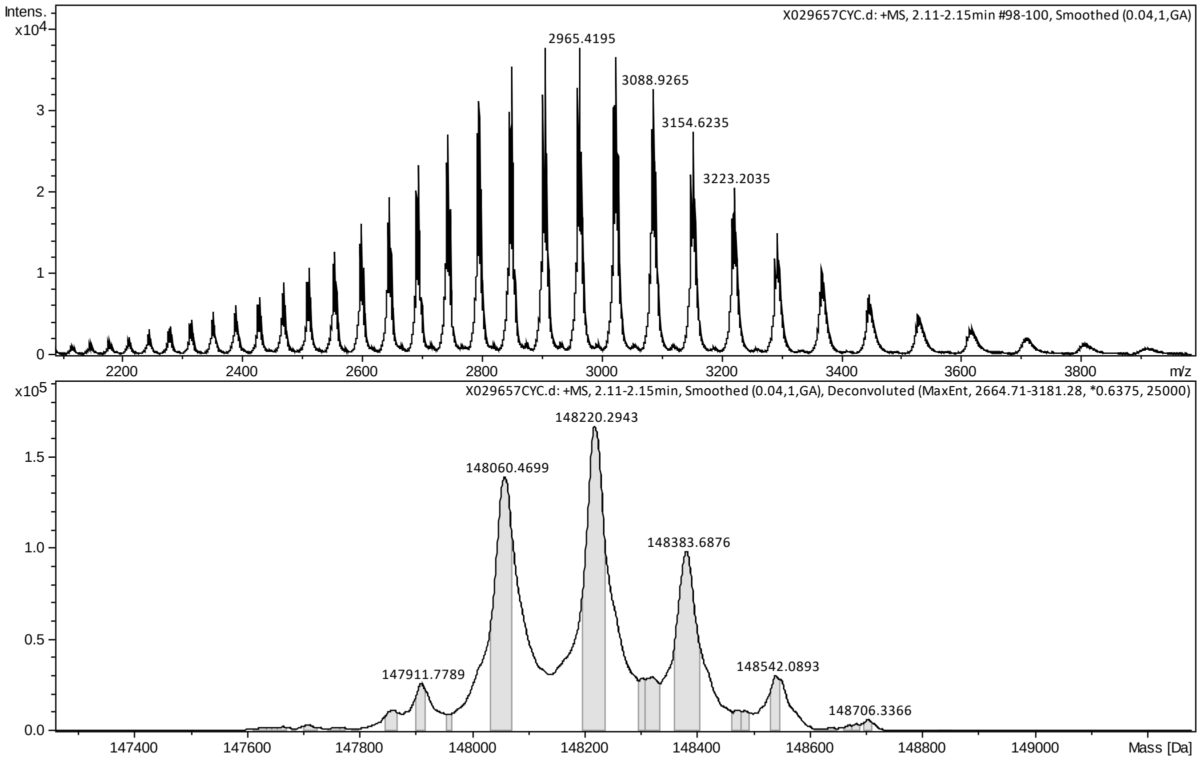
Intens.
x10^4
X029657CYC.d: +MS, 2.11-2.15min #98-100, Smoothed (0.04,1,GA)
2965.4195
3088.9265
3154.6235
3223.2035
m/z
x10^5
X029657CYC.d: +MS, 2.11-2.15min, Smoothed (0.04,1,GA), Deconvoluted (MaxEnt, 2664.71-3181.28, *0.6375, 25000)
148220.2943
148060.4699
148383.6876
147911.7789
148542.0893
148706.3366
Mass [Da]

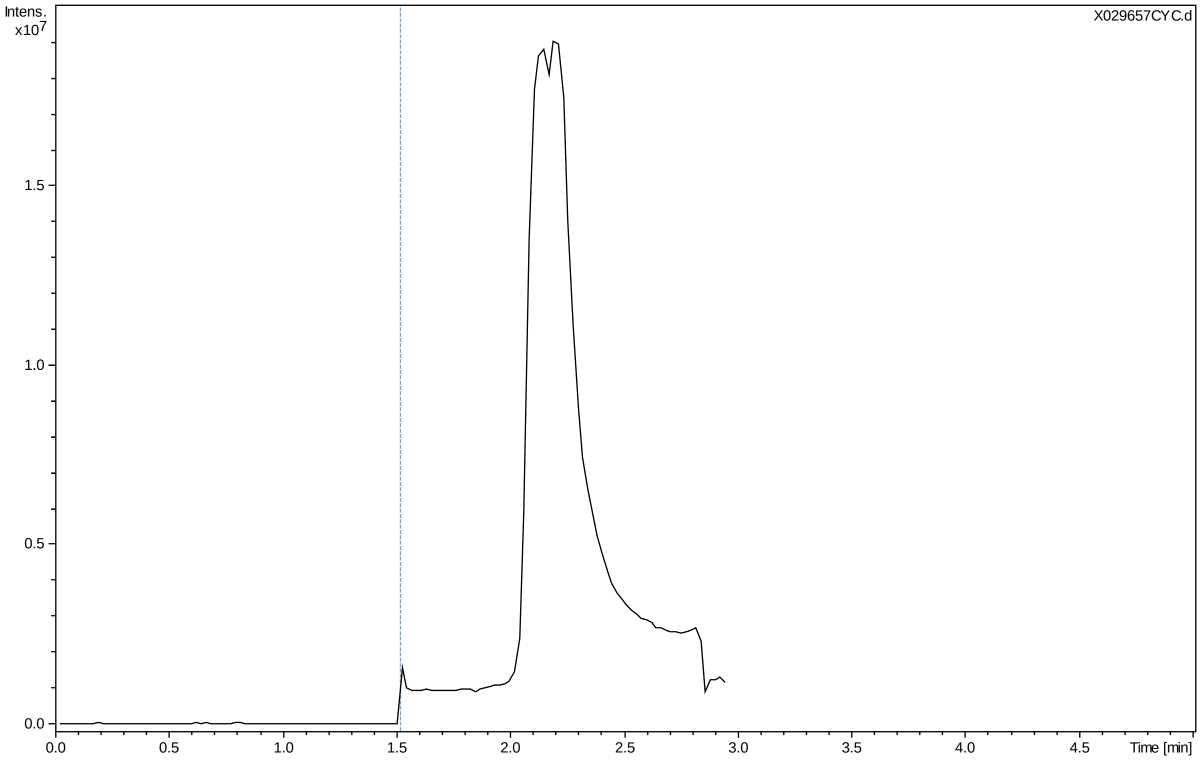
Intens.
x10^7
X029657CYC.d
Time [min]

Deglycosylated TTZ

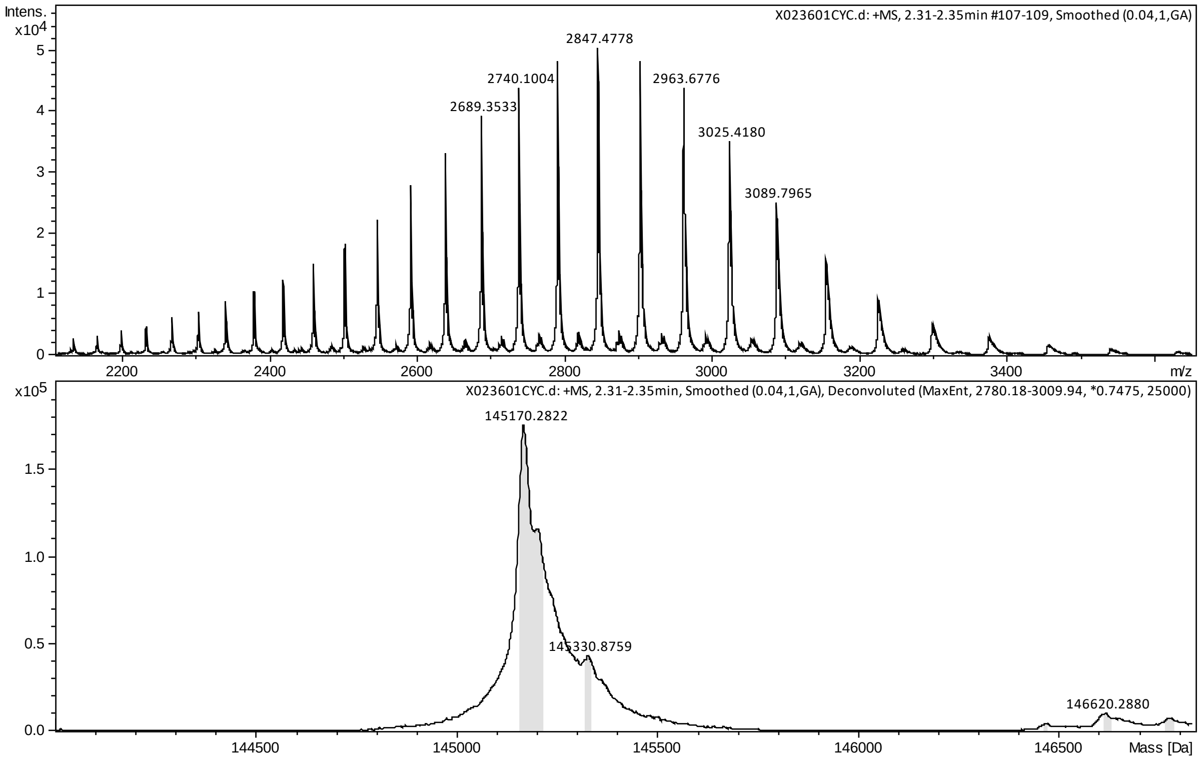


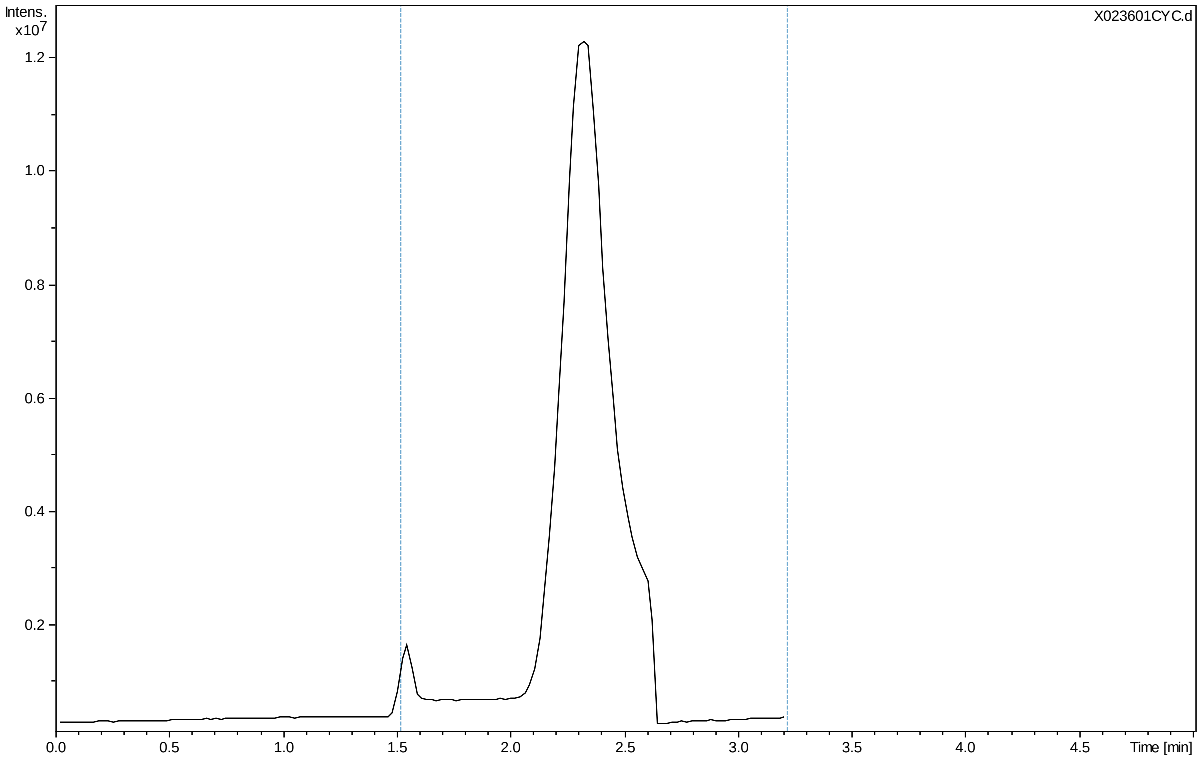

TTZ-SGM-BDP **7a**

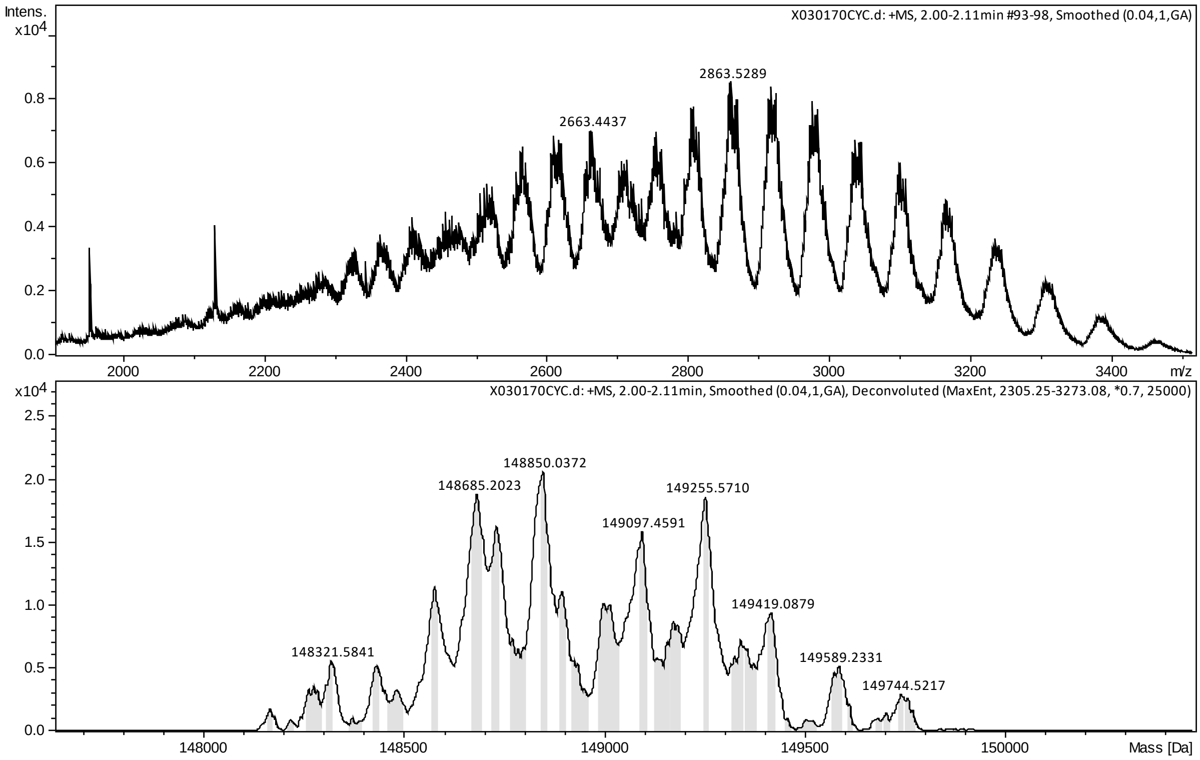


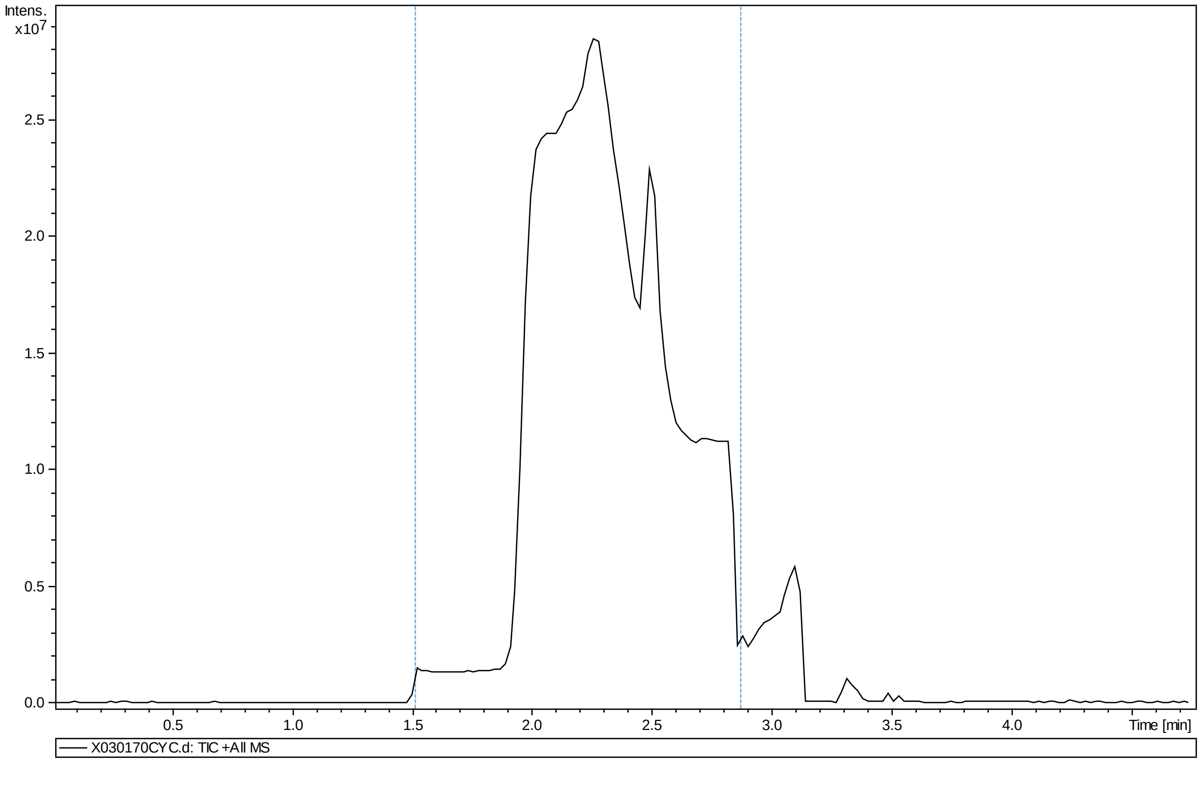

TTZ-SGM-FLU **7b**

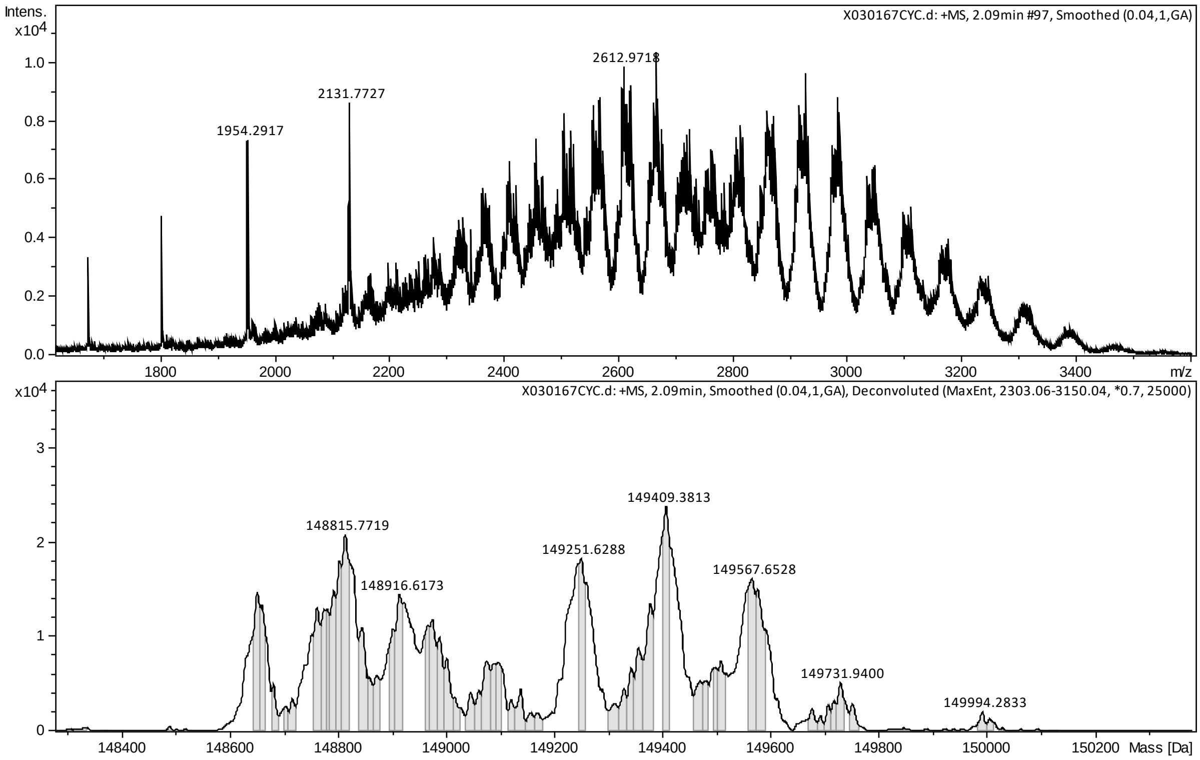


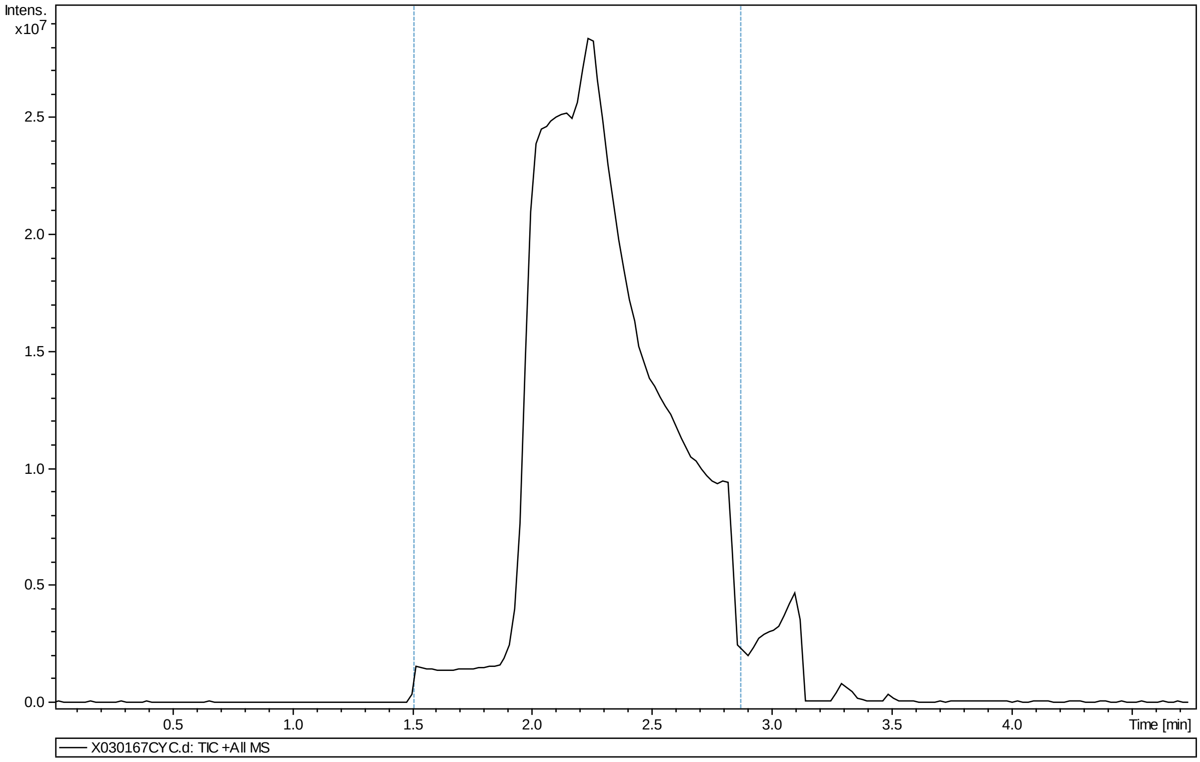

RTX-SGM-BDP **8a**

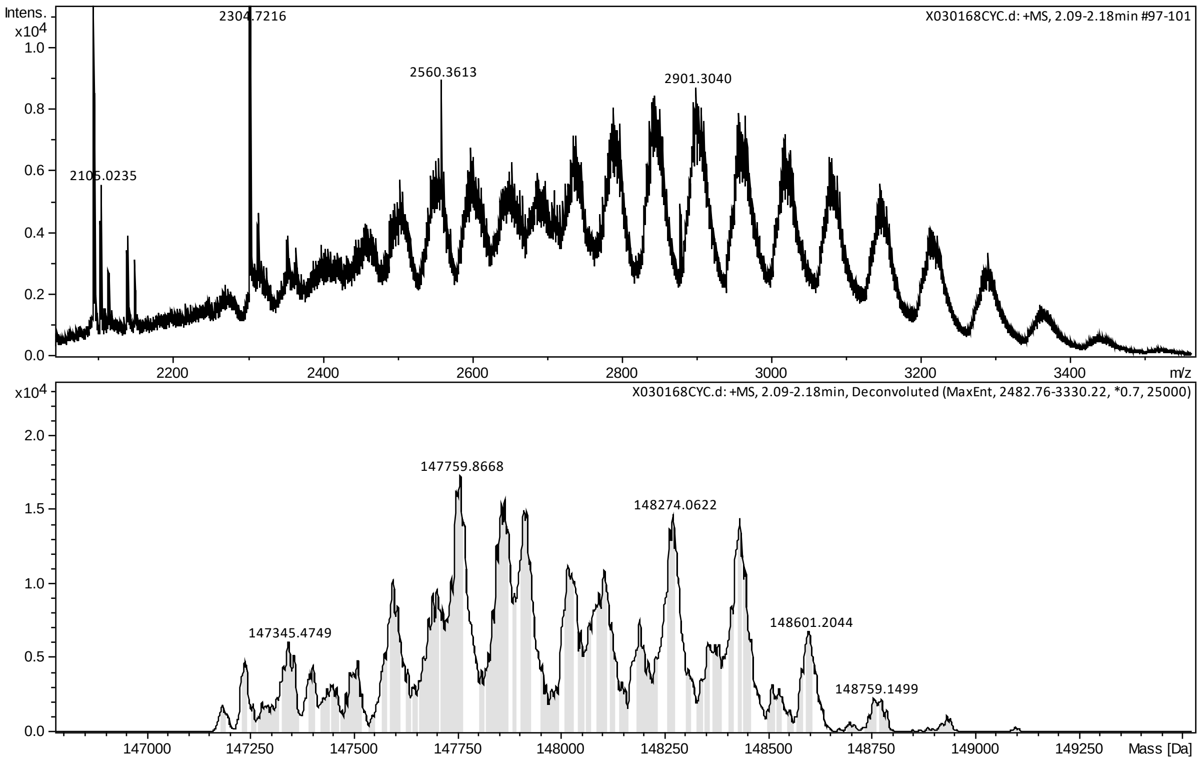


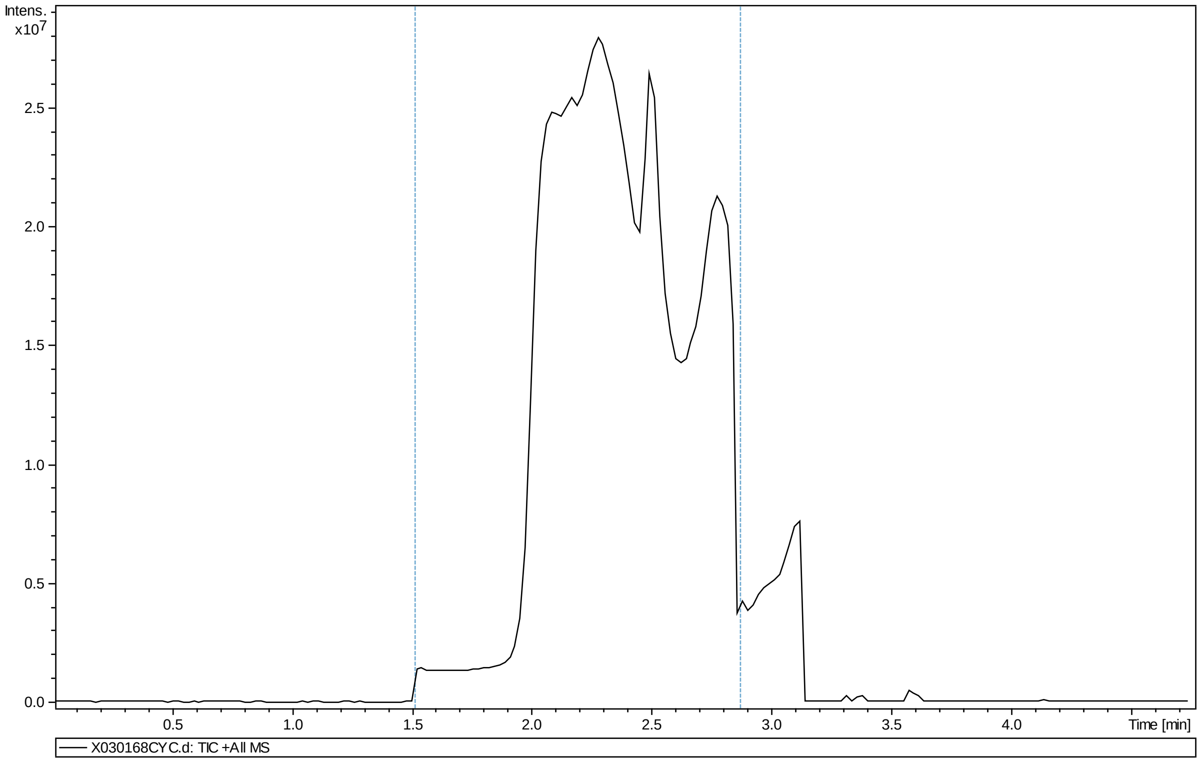

RTX-SGM-FLU **8b**

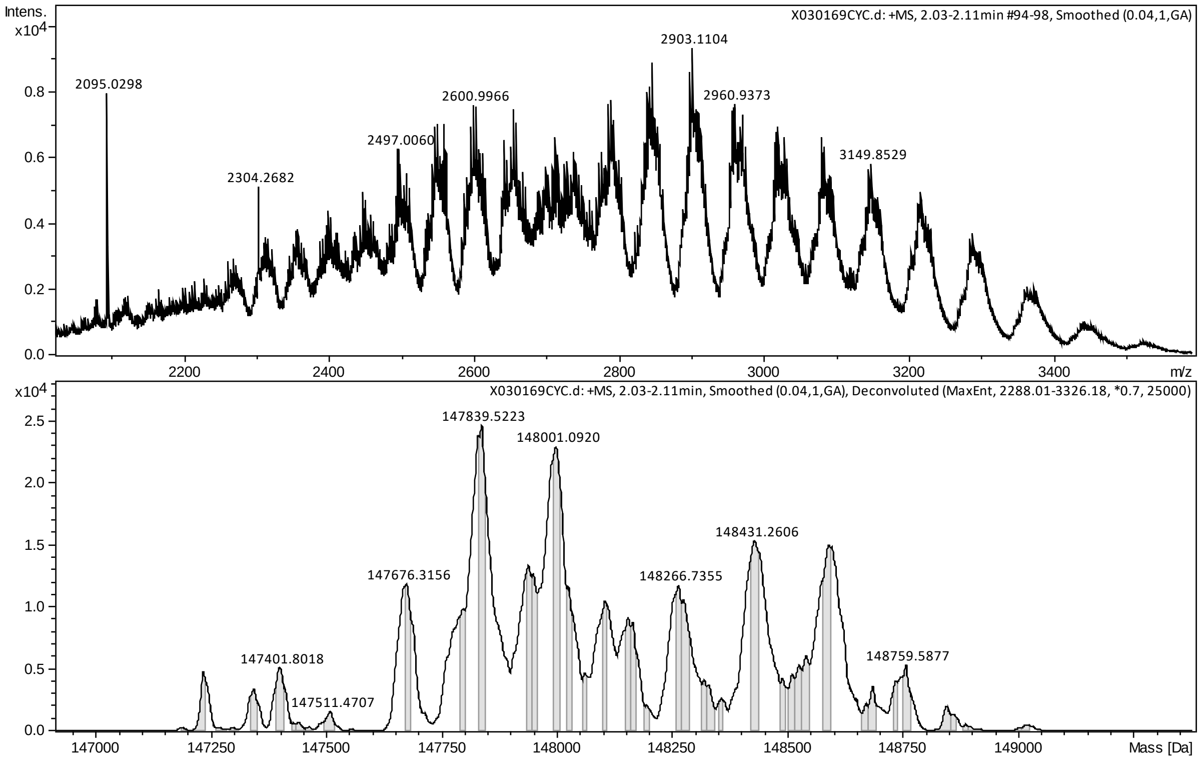


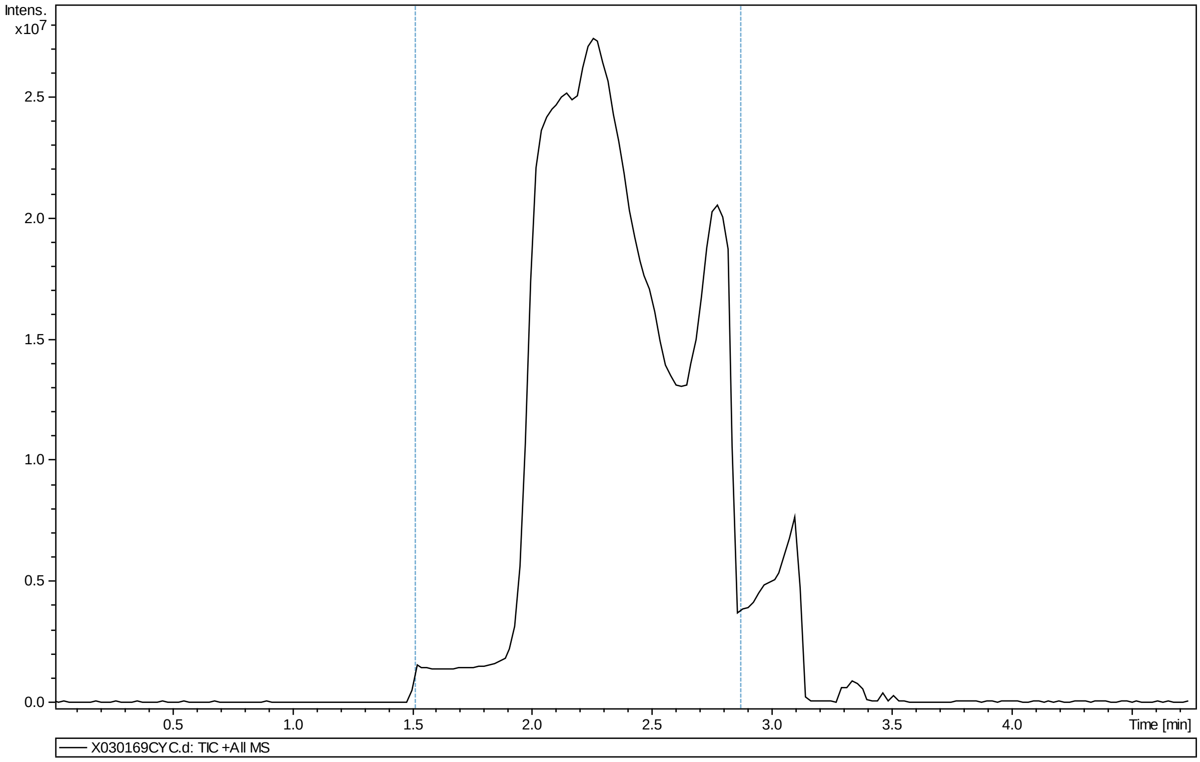

TTZ-BDP **9a**

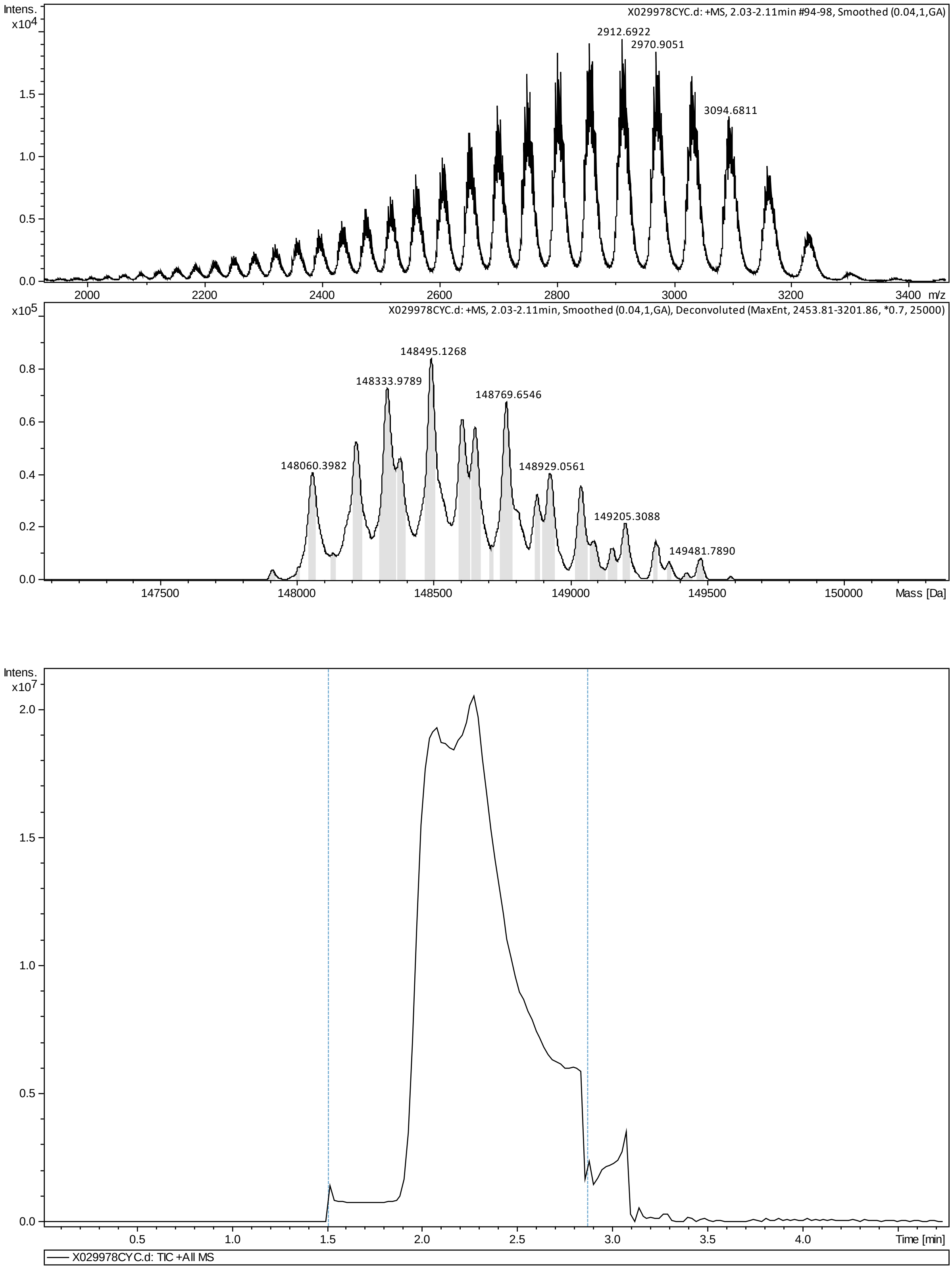

Intens. x10^4
X029978CYC.d: +MS, 2.03-2.11min #94-98, Smoothed (0.04,1,GA)
2912.6922
2970.9051
3094.6811
m/z
x10^5
X029978CYC.d: +MS, 2.03-2.11min, Smoothed (0.04,1,GA), Deconvoluted (MaxEnt, 2453.81-3201.86, *0.7, 25000)
148495.1268
148333.9789
148769.6546
148060.3982
148929.0561
149205.3088
149481.7890
Mass [Da]
Intens. x10^7
Time [min]
X029978CYC.d: TIC +All MS

# TTZ-FLU **9b**

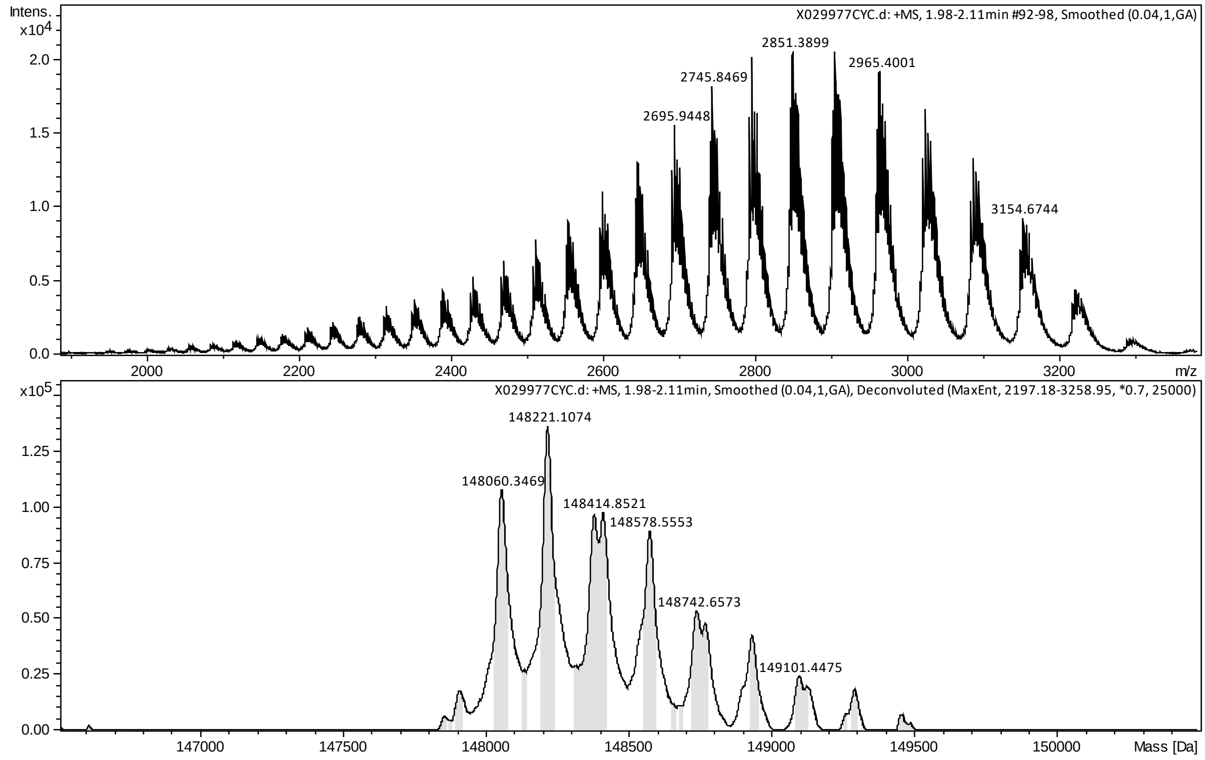
Intens.
x10^4
X029977CYC.d: +MS, 1.98-2.11min #92-98, Smoothed (0.04,1,GA)
2851.3899
2965.4001
2745.8469
2695.9448
3154.6744
m/z
X029977CYC.d: +MS, 1.98-2.11min, Smoothed (0.04,1,GA), Deconvoluted (MaxEnt, 2197.18-3258.95, *0.7, 25000)
x10^5
148221.1074
148060.3469
148414.8521
148578.5553
148742.6573
149101.4475
Mass [Da]

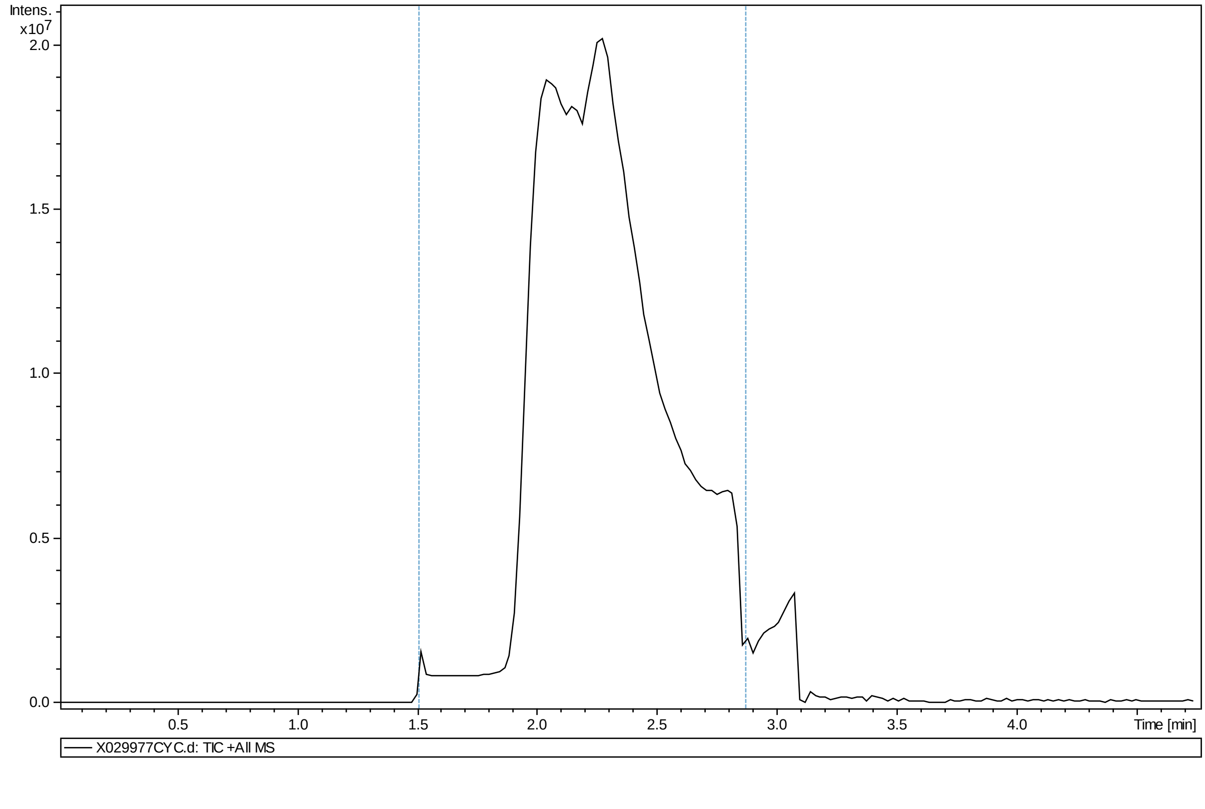
Intens.
x10^7
Time [min]
X029977CYC.d: TIC +All MS

RTX-BDP **10a**

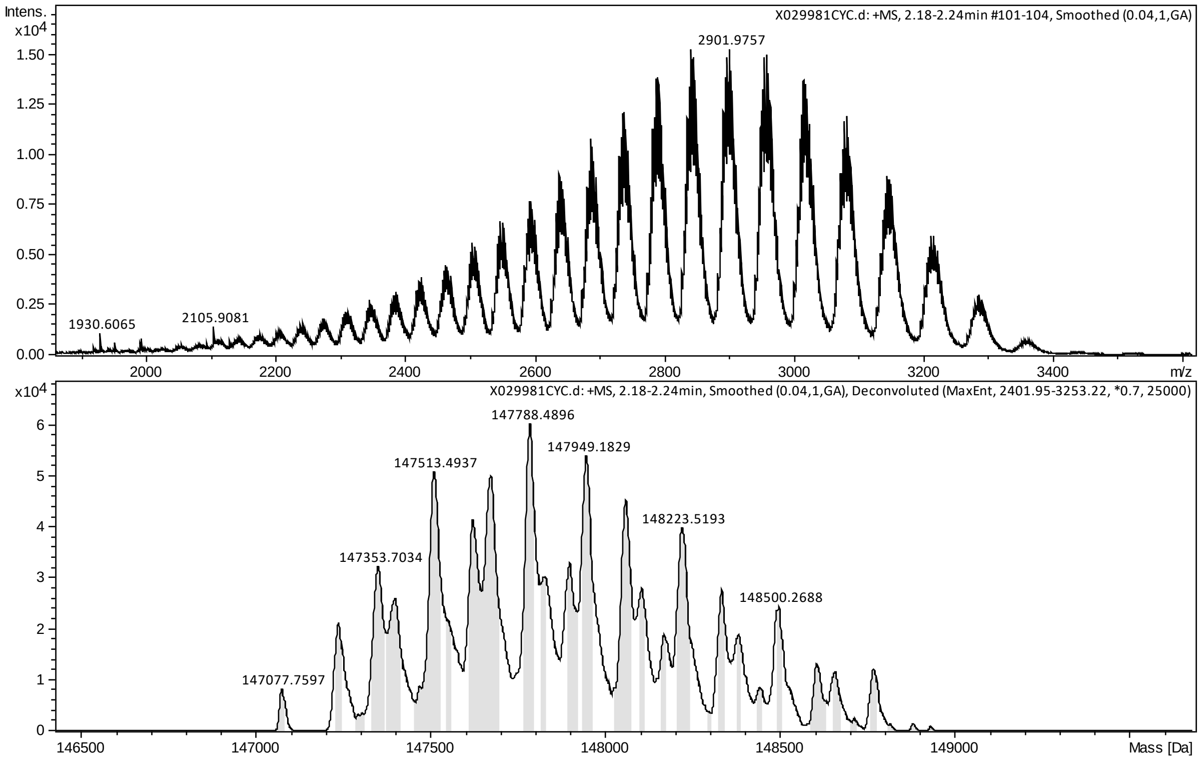

X029981CYC.d: +MS, 2.18-2.24min #101-104, Smoothed (0.04,1,GA)
2901.9757
1930.6065
2105.9081
X029981CYC.d: +MS, 2.18-2.24min, Smoothed (0.04,1,GA), Deconvoluted (MaxEnt, 2401.95-3253.22, *0.7, 25000)
147788.4896
147949.1829
147513.4937
148223.5193
147353.7034
148500.2688
147077.7597
Intens. x10^4
m/z
Mass [Da]


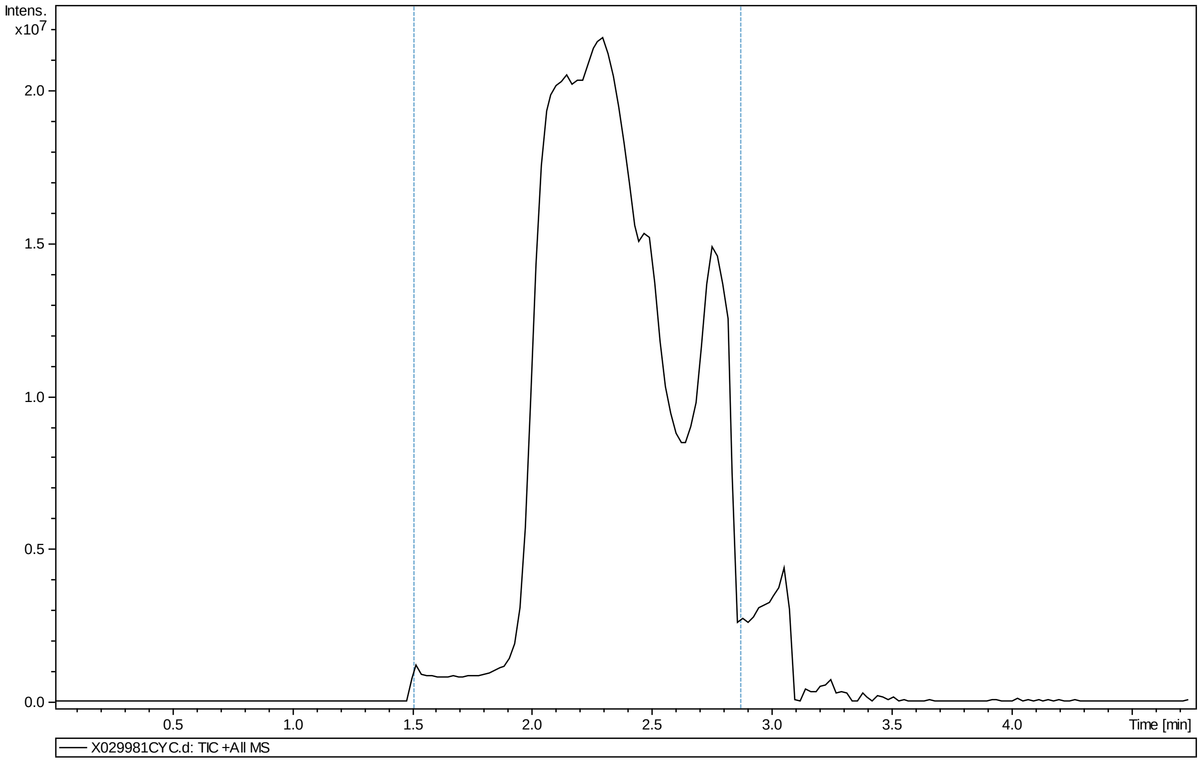

Intens. x10^7
Time [min]
X029981CYC.d: TIC +All MS

RTX-FLU **10b**

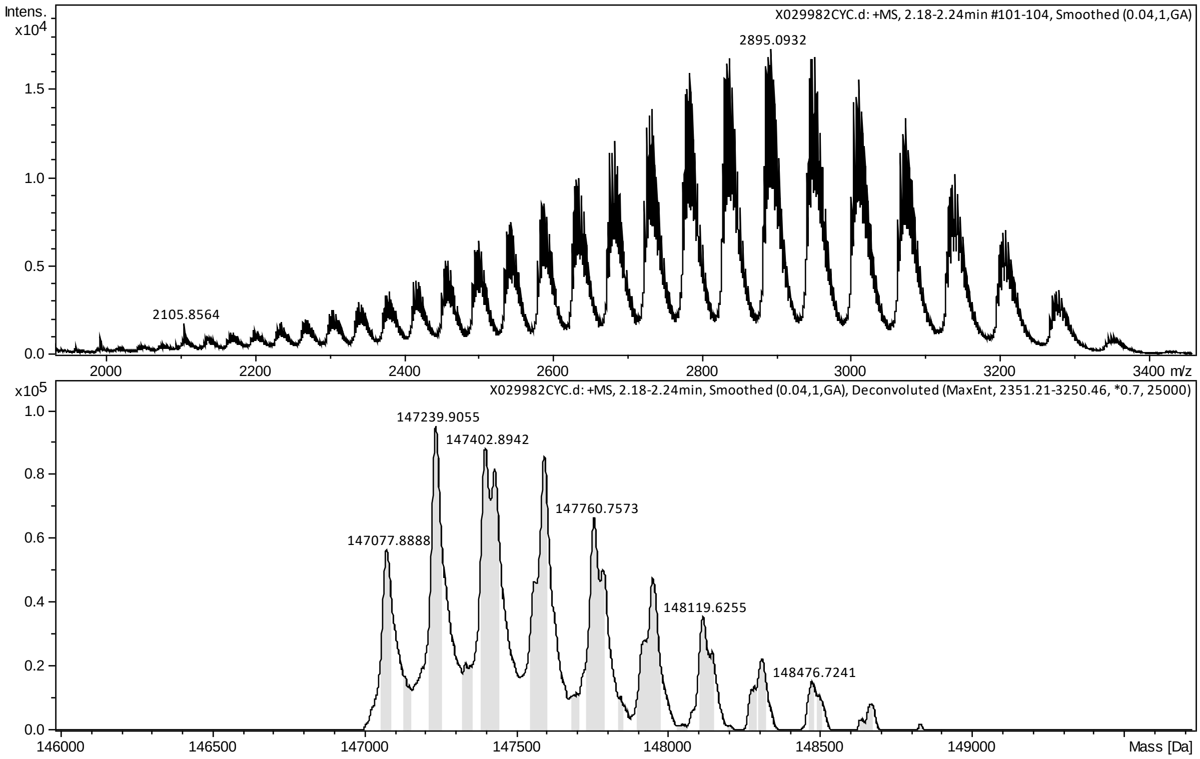
Intens.
x10^4
X029982CYC.d: +MS, 2.18-2.24min #101-104, Smoothed (0.04,1,GA)
2895.0932
2105.8564
1.5
1.0
0.5
0.0
2000
2200
2400
2600
2800
3000
3200
3400 m/z
x10^5
X029982CYC.d: +MS, 2.18-2.24min, Smoothed (0.04,1,GA), Deconvoluted (MaxEnt, 2351.21-3250.46, *0.7, 25000)
147239.9055
147402.8942
147760.7573
147077.8888
148119.6255
148476.7241
1.0
0.8
0.6
0.4
0.2
0.0
146000
146500
147000
147500
148000
148500
149000
Mass [Da]

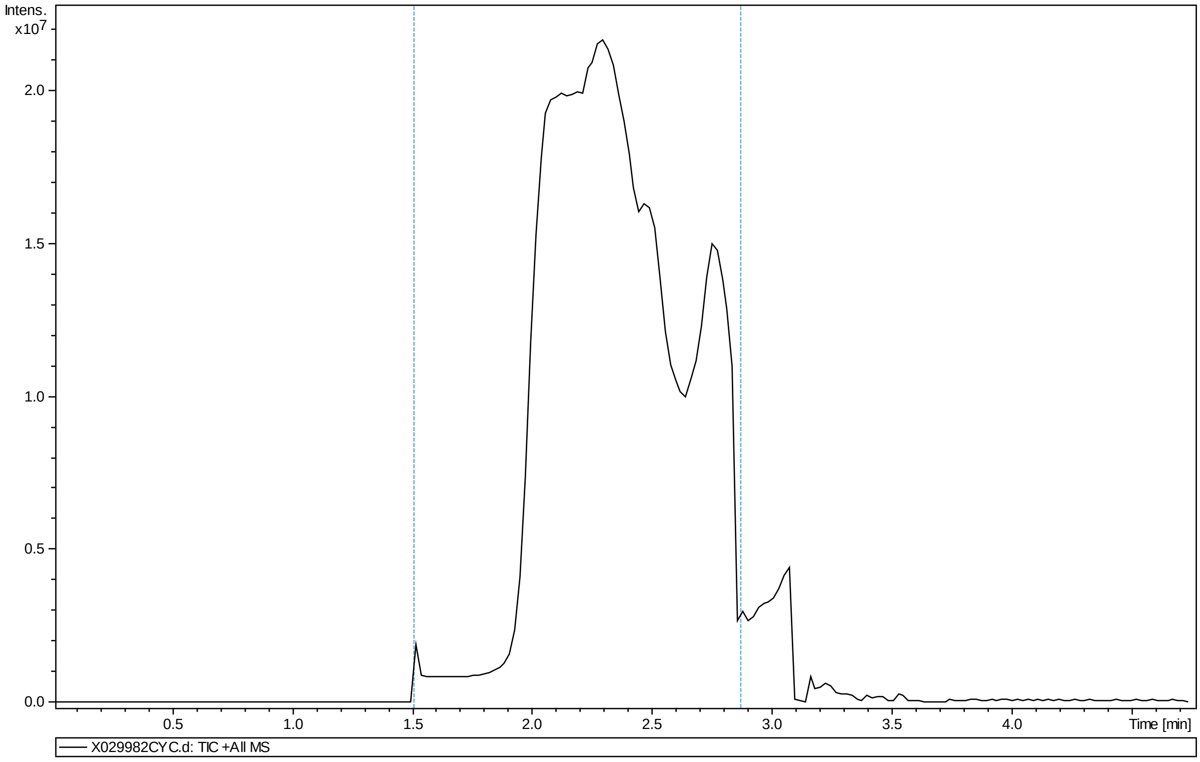
Intens.
x10^7
2.0
1.5
1.0
0.5
0.0
0.5
1.0
1.5
2.0
2.5
3.0
3.5
4.0
Time [min]
X029982CYC.d: TIC +All MS

**No native mAb remains after labeling using cross-linking technology, e.g.:**

**Native RTX (red) vs RTX-SGM-FLU (black)**

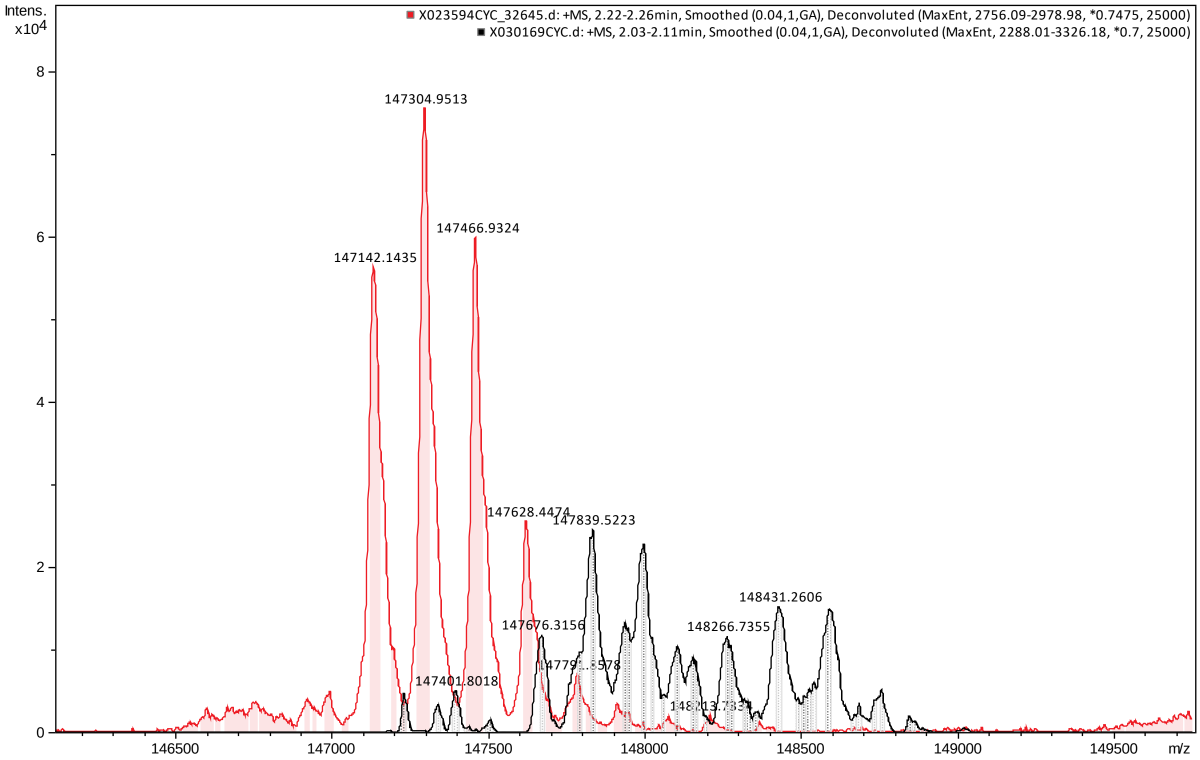

To study antigen binding, we decided to focus on fluorescein-labelled AFCs **7b**, **8b**, **9b** and **10b**, considering their higher fluorescence emission and the strong quenching of BODIPY among AFCs TTZ-SGM-BDP **7a** and RTX-SGM-BDP **8a**. First, HER2 affinities of AFCs TTZ-SGM-FLU **7b** and TTZ-FLU **9b** were determined by ELISAs (**Figure S4**), between 0.01 nM and 31 nM. No difference in binding affinity between the two species was observed, with similar affinity to HER2 for AFC generated through either lysine or cross-linked conjugation.

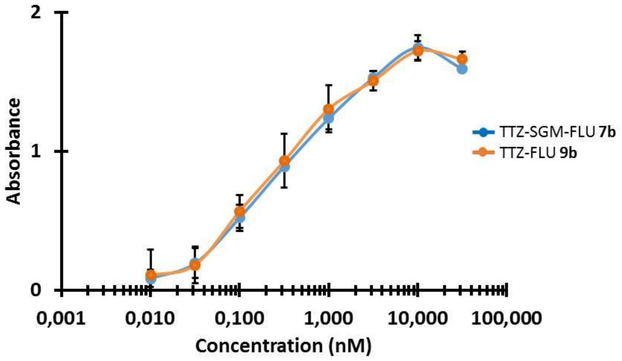


**Figure S4.** HER2 affinities of AFCs TTZ-SGM-FLU **7b** (blue curve) and TTZ-FLU **9b** (orange curve) by ELISAs.

Then, binding of AFCs RTX-SGM-FLU **8b** and RTX-FLU **10b** to CD20 were assessed using flow cytometry (**Figure S5**). Considering their fluorescence emission data (**Figure 2B**), it is possible to compare directly the binding to CD20 antigen expressed on Daudi cells *via* fluorescence ratios (**Figure S5**). Flow cytometry experiments, conducted between 1 and 100 µg/mL of protein, showed a significative higher fluorescence ratio for AFC RTX-SGM-FLU **8b** compared to AFC RTX-FLU **10b** at pH 7 (**Figure S5**). Hence, AFC RTX-SGM-FLU **8b** had a higher binding to CD20 than AFC RTX-FLU **10b** at pH 7, which could be linked to several lysine residues very close to the CDRs of RTX (**Figure S6**). Moreover, acidification to pH 6, while reducing the fluorescence ratio of both AFCs, did not change their relative affinity to CD20 (**Figure S7**). Affinity could not be assessed in these conditions, despite important AFC concentrations. This is due to operating conditions, namely the cell concentration used for flow cytometry, which needed to be high enough for fast analysis, and the high CD20 expression at the surface of these cells, keeping us back from saturation.

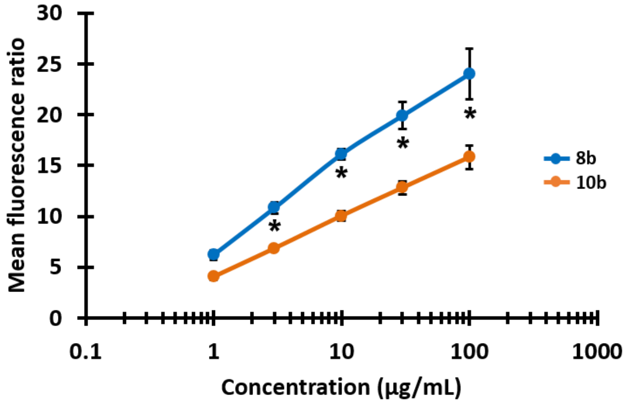


**Figure S5.** Binding of AFCs RTX-SGM-FLU **8b** (blue curve) and RTX-FLU **10b** (orange curve) to CD20 by flow cytometry.

**Table S4.** Statistical analysis on CD20 binding

| | | Concentration (µg/mL) | | | | |
|---|---|---|---|---|---|---|
| | | 100 | 30 | 10 | 3 | 1 |
| pH6 | RTX-SGM-FLU **8b** | 0.1 (n.s.) | 0.4 (n.s.) | 0.4 (n.s.) | 0.2 (n.s.) | 0.4 (n.s.) |
| | RTX-FLU **10b** | | | | | |
| pH7 | RTX-SGM-FLU **8b** | 0.029 (*) | 0.029 (*) | 0.029 (*) | 0.029 (*) | 0.029 (*) |
| | RTX-FLU **10b** | | | | | |

A Wilcoxon rank sum test was used. The level of significance was set at 5% (two-sided).

p-Values of AFCs at pH 7 and pH 6 are presented.

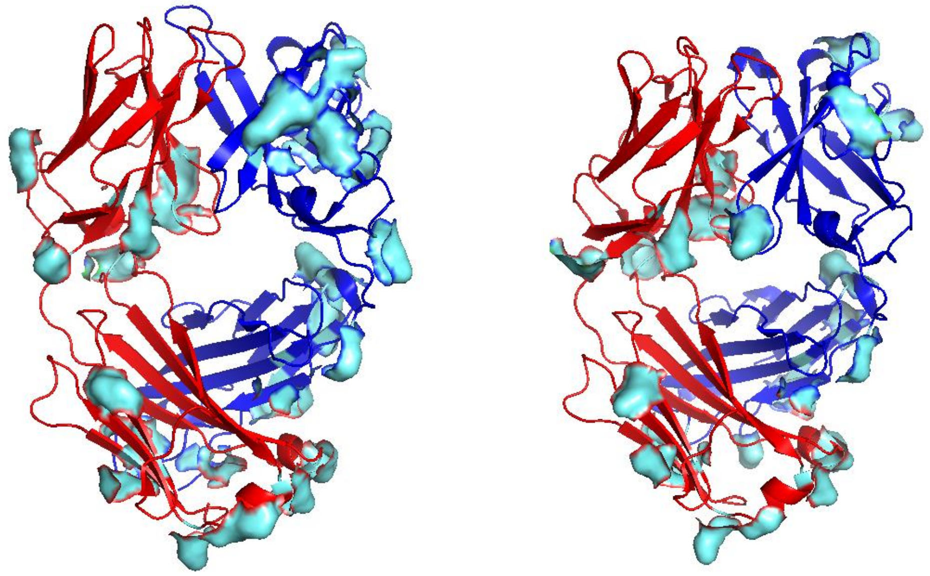

**Figure S6.** Models representing rituximab (left) and trastuzumab (right), from PDB structures 2OSL and 4HKZ respectively. Heavy (blue) and light (red) chains are represented as cartoons, and lysines are visualized as cyan surfaces. In the V-domains, trastuzumab contains 9 lysine residues (4 in VH and 5 in VL), among which one is contained inside CDRH1 and 2 others in the heavy chain framework are close to the paratope. For rituximab, the V-domains include 12 lysine residues among which 6, all inside the heavy chain, are very close to the paratope.

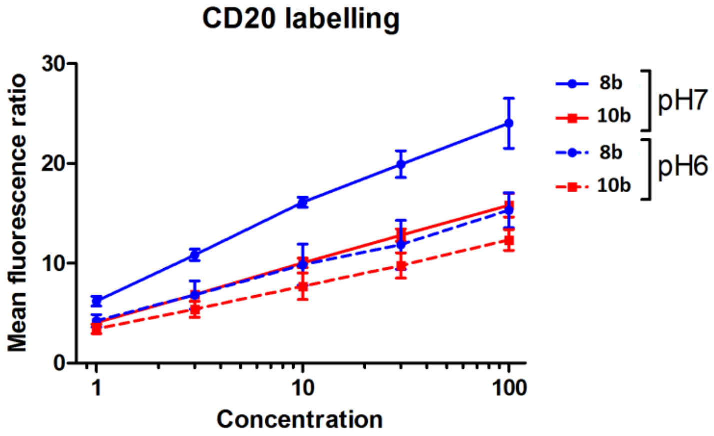


**Figure S7.** Flow cytometry of AFCs RTX-SGM-FLU **8b** and RTX-FLU **10b** at pH7 and pH6, n = 3

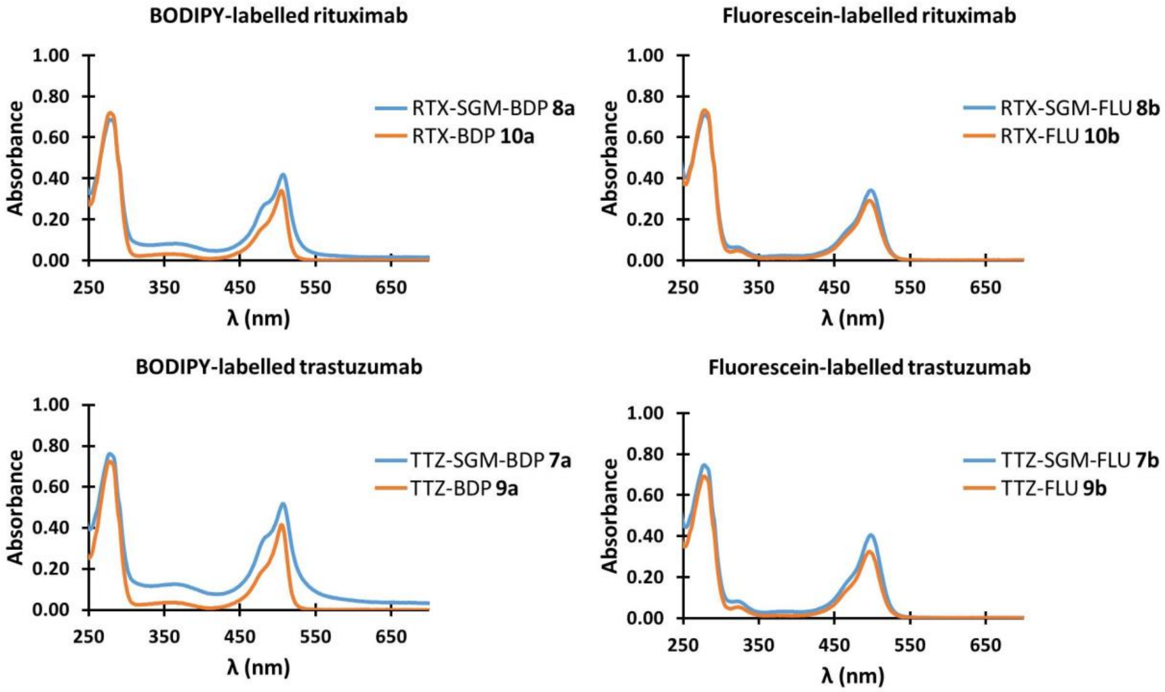


**Figure S8.** UV-visible absorption of AFCs. Sample concentration was adjusted to 500 µg/mL of protein.

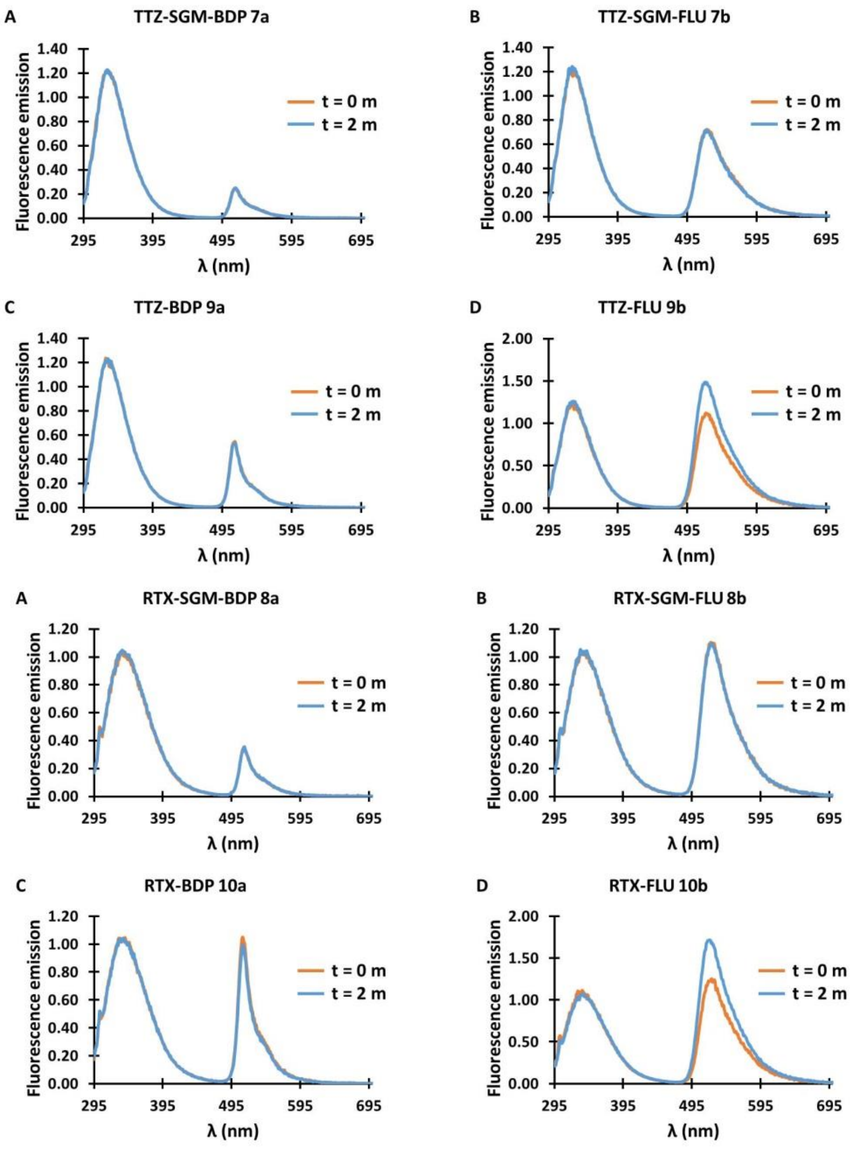


**Figure S9.** Fluorescence emission of fresh (orange curves) and stored in the dark at 4 °C (blue curves) AFCs based on rituximab after normalisation. Excitation wavelength: 275 nm. For easier comparison, the sample concentration was adjusted to 50 µg/mL of protein.

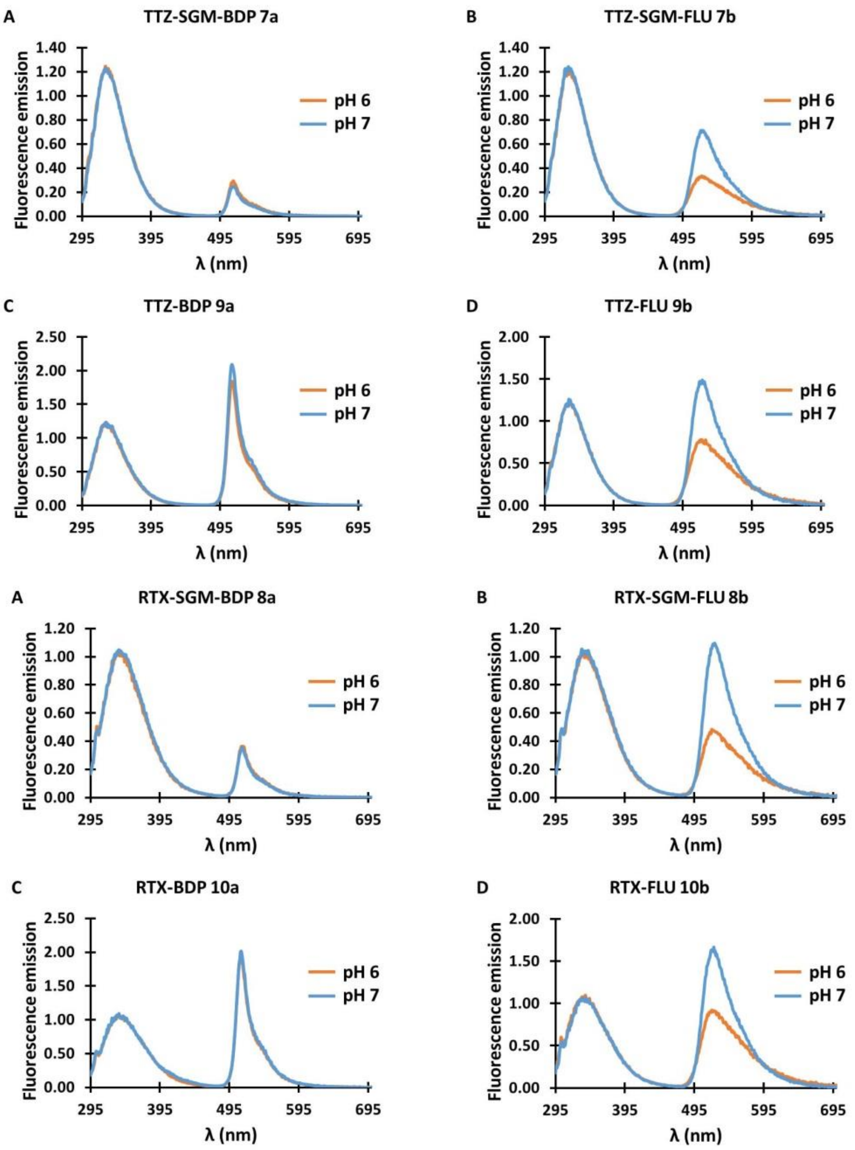


**Figure S10.** Comparison of fluorescence emission of AFCs based on rituximab at pH 7 (blue curves) and 6 (orange curves) after normalisation. Excitation wavelength: 275 nm. For easier comparison, the sample concentration was adjusted to 50 µg/mL of protein.

## References

(1) Castañeda, L., Wright, Z. V. F., Marculescu, C., Tran, T. M., Chudasama, V., Maruani, A., Hull, E. A., Nunes, J. P. M., Fitzmaurice, R. J., Smith, M. E. B., Jones, L. H., Caddick, S., and Baker, J. R. (2013) A mild synthesis of N-functionalised bromomaleimides, thiomaleimides and bromopyridazinediones. *Tetrahedron Lett. 54*, 3493–3495.

(2) Suzuki, T., Hisakawa, S., Itoh, Y., Suzuki, N., Takahashi, K., Kawahata, M., Yamaguchi, K., Nakagawa, H., and Miyata, N. (2007) Design, synthesis, and biological activity of folate receptor-targeted prodrugs of thiolate histone deacetylase inhibitors. *Bioorganic Med. Chem. Lett. 17*, 4208–4212.
